# Bioactive Silages from Agro-Industrial By-Products Based on Grape Pomace or Olive Mill Wastewater for Ruminants: Evolution of Phenolic Profiles, Antioxidant Activity, and Fatty Acid Composition

**DOI:** 10.3390/antiox15060692

**Published:** 2026-05-30

**Authors:** Roberta Savina Dibenedetto, Mónica Sánchez-Parra, José Luis Ordóñez-Díaz, Alessio Di Luca, Giovanni Martemucci, José Manuel Moreno-Rojas, Angela Gabriella D’Alessandro

**Affiliations:** 1 Department of Soil, Plant and Food Sciences, University of Bari, 70126 Bari, Italy; roberta.dibenedetto@uniba.it (R.S.D.); alessio.diluca@uniba.it (A.D.L.); gmartem@libero.it (G.M.); 2 Department of Agroindustry and Food Quality, Andalusian Institute of Agricultural and Fisheries Research and Training (IFAPA) Alameda del Obispo, Avda. Menéndez–Pidal S/N., 14004 Córdoba, Spain; monica.sanchez.parra@juntadeandalucia.es (M.S.-P.); josel.ordonez@juntadeandalucia.es (J.L.O.-D.); 3 Programa de Doctorado en Ingeniería Agraria, Alimentaria, Forestal y de Desarrollo Rural Sostenible, Universidad de Córdoba, 14071 Córdoba, Spain

**Keywords:** functional silage, agro-industrial by-products, grape pomace, olive mill wastewater, polyphenolic profile, antioxidant capacity, ruminant feeding

## Abstract

This study investigated the chemical composition, fermentation dynamics, fatty-acid profile, and polyphenolic evolution of two mixed silages designed to valorize agro-industrial by-products for ruminant feeding. Silages were produced by co-ensiling wheat straw, cheese-whey, and molasses with grape pomace (SIL-1) or olive mill wastewater (SIL-2), and were monitored over a 150-day ensiling period. The two formulations exhibited distinct compositional characteristics and fermentation kinetics. SIL-1 showed higher crude protein content and a more favorable fatty-acid profile, with greater levels of selected long-chain fatty acids, whereas SIL-2 had higher dry matter and structural fiber fractions. Both silages achieved effective fermentation, reaching stable acidic conditions (pH < 4.0), although SIL-1 consistently maintained lower pH and higher buffering capacity. Fermentation end-products differed between silages, with higher concentrations of short-chain fatty acids in SIL-1 and greater lactic acid accumulation in SIL-2, under significant treatment × time interactions. Bioactive compound analysis revealed higher total phenolic content and antioxidant capacity in SIL-1, whereas SIL-2 showed marked degradation of phenolic compounds, including the loss of characteristic secoiridoids. Polyphenolic profiles displayed compound-specific temporal dynamics during ensiling. Overall, both silages were well preserved; however, SIL-1 demonstrated superior nutritional quality and bioactive stability, supporting its potential as a functional feed ingredient for ruminant nutrition.

## 1. Introduction

In Europe, the agri-food sector produces vast quantities of by-products each year, generating significant environmental and economic challenges. According to Eurostat, more than 59 million tonnes of food waste were generated in the European Union in 2022, with approximately 19% originating from food processing and manufacturing stages [1,2]. Among agri-food chains, the olive oil, wine, cereal, and dairy industries represent major contributors to the generation of these by-products. When inadequately managed, by-products such as olive mill wastewater (OMWW) and grape pomace (GP) contribute to greenhouse gas emissions, soil and water pollution, and high disposal costs [3,4,5].

OMWW is a liquid by-product generated during the mechanical extraction of olive oil. Worldwide production is estimated to range from 10 to more than 30 million m^3^ per year, with typical yields of approximately 1.2 m^3^ per tonne of olives processed [3,5]. OMWW is characterized by a very high organic load and by elevated concentrations of polyphenolic compounds that confer strong antioxidant properties but are also phytotoxic and poorly biodegradable [3,4].

GP, the main solid by-product of winemaking, is produced in quantities estimated between 10.5 and 13.1 million annual tonnes, reflecting the global scale of wine production. Europe is the largest contributor, with Italy, France, and Spain accounting for approximately half of worldwide wine output [6,7]. GP contains substantial amounts of structural carbohydrates, organic acids, residual sugars, and phenolic compounds, which enhance its functional value while also limiting its direct disposal due to slow biodegradation and potential environmental impact [8,9].

The magnitude and the pronounced seasonality of OMWW and GP production highlight the urgent need for effective stabilization and valorization strategies. Within a waste-to-resource framework, their recycling constitutes a sustainable strategy for mitigating the environmental burden. Such an approach, designed to facilitate the transition toward a circular economy model, can reduce pollution, promote resource efficiency, and contribute to environmental protection, particularly in Mediterranean regions where olive oil and wine production are geographically concentrated.

A distinctive feature of these by-products is their richness in bioactive compounds, especially polyphenols, which have attracted increasing interest due to their antioxidant, antimicrobial, and anti-inflammatory properties, with documented benefits in both human and animal nutrition [10,11,12,13]. The total polyphenol content of OMWW typically ranges from 0.3–0.4 g L^−1^ in untreated samples [3,14,15], and may increase up to approximately 2.5 g L^−1^ in acidified extracts [16]. GP is similarly recognized as a rich source of phenolics, with reported values of 144–298 mg gallic acid equivalents (GAE) g^−1^ dry matter (DM) in skins and 327–540 mg GAE g^−1^ DM in seeds [17,18].

In recent years, the use of GP as a feed ingredient has been widely addressed in the context of agro-industrial by-product valorization in animal nutrition. Recent reviews consistently indicate that GP can be incorporated into ruminant diets at moderate inclusion levels without detrimental effects on animal performance, while potentially improving the antioxidant status and lipid composition of milk and meat [7,19].

Similarly, olive-derived byproducts obtained from OMWW have been explored as dietary supplements in ruminants and other livestock species, with reported positive effects of OMWW-derived phenolic compounds—particularly hydroxytyrosol and related secoiridoids—on redox balance and antioxidant capacity, without adverse effects on rumen fermentation, nutrient utilization, or productivity when administered at controlled doses [20,21].

In this context, effective valorization strategies for these by-products require technological approaches that stabilize chemically complex agro-industrial matrices while preserving their functional properties over time.

A major constraint to the direct use of raw by-products in ruminant feeding is their marked seasonality and perishability, which hinder continuous and standardized supply. Ensiling suitable mixtures of agro-industrial byproducts has therefore emerged as a practical and sustainable strategy to stabilize seasonal availability and facilitate on-farm use [22,23]. Silage is a well-established preservation technique based on controlled anaerobic fermentation, allowing year-round feed availability and increased dietary flexibility through the inclusion of heterogeneous raw materials [24]. When properly managed, ensiled feeds have been associated with improved nutrient utilization, digestibility, and productive performance in ruminants [25].

In fact, it is well known that under anaerobic conditions of the ensiling process, lactic fermentation rapidly lowers pH, inhibits spoilage microorganisms, and contributes to preserving nutritional quality. However, factors such as composition of the ingredients, moisture content, buffering capacity, and fermentation kinetics affect the silage quality and its suitability for ruminant feeding [23,26,27], factors that become particularly critical when complex mixtures rich in phenolic compounds are ensiled. In this regard, integrating OMWW and GP together with other agricultural byproducts such as straw and cheese whey into mixed silages could, therefore, extend shelf life and support sustainable feeding strategies.

Beyond their direct dietary application, GP and olive-derived by-products have also been evaluated within silage systems. Available studies indicate that their inclusion in silage formulations does not negatively affect fermentation quality or animal performance when applied at moderate inclusion levels [28,29]. Moreover, mixed silage systems combining GP, OMWW, and other byproducts have shown adequate fermentation characteristics and stability, supporting their practical applicability [28,30].

However, despite these promising findings, information on the optimization of co-ensiling strategies involving heterogeneous agro-industrial by-products remains limited, particularly with respect to the combined use of GP and OMWW under farm-scale conditions. The high variability in chemical composition and phenolic content of these substrates may influence fermentation dynamics, nutrient availability, and microbial activity, highlighting the need for targeted studies focusing on the stability of bioactive compounds and the functional quality of silages obtained from complex by-product mixtures.

In a previous laboratory-scale study, we demonstrated that ensiling raw mixed by-products (straw, GP, OMWW, and cheese whey), without preliminary fractionation or processing, was technically feasible and resulted in nutritionally stable silages retaining antioxidant properties over time, constituting a potential source of antioxidant compounds for ruminants [28].

Building on these findings, the present study aimed to further explore integrated, multi-by-product silage systems, combining either GP or OMWW with wheat straw and cheese whey, by evaluating their quality under farm-scale silage conditions (≈300 kg bales). Chemical composition, fermentation parameters, and antioxidant capacity were monitored over a 150-day maturation period. The working hypothesis was that these formulations could produce nutritionally stable feed while retaining significant antioxidant potential over time, making them suitable for sustainable ruminant nutrition.

## 2. Materials and Methods

### 2.1. Chemicals

Methanol (HPLC grade, ≥99.9%), chloroform (≥99%), n-hexane (≥99%), diethyl ether (≥99%), formic acid (≥98%), phosphoric acid (85%), sulfuric acid (≥98%), sodium hydroxide (≥98%), potassium hydroxide (≥98%), and glacial acetic acid (≥99%) were purchased from Merck KGaA (Darmstadt, Germany). Trolox (6-hydroxy-2,5,7,8-tetramethylchroman-2-carboxylic acid, ≥97%), 2,2-diphenyl-1-picrylhydrazyl (DPPH, ≥95%), 2,2′-azino-bis(3-ethylbenzothiazoline-6-sulfonic acid) diammonium salt (ABTS, ≥98%), fluorescein sodium salt (≥95%), 2,2′-azobis(2-amidinopropane) dihydrochloride (AAPH, ≥98%), Folin–Ciocalteu reagent, gallic acid (≥98%), nonadecanoic acid (C19:0, ≥99%), and standard lactic acid (≥98%) were obtained from Sigma-Aldrich (Steinheim, Germany). Ammonium chloride (≥99%), phenol (≥99%), sodium hypochlorite (≥98%), and potassium persulfate (≥98%) were purchased from VWR International Eurolab S.L. (Barcelona, Spain), while sodium carbonate (≥99%), potassium phosphate monobasic/dibasic (≥99%), and acetic acid (≥99.8%) were supplied by PanReac AppliChem (Barcelona, Spain).

The short-chain fatty acid (SCFA) standard mix (TraceCERT^®^, CRM46975, 10 mM in water) and the 37-component fatty acid methyl ester (FAME) mix (CRM47885) were purchased from Supelco (Bellefonte, PA, USA). Reference phenolic and antioxidant standards, including catechin, epicatechin, caffeic acid, ferulic acid, rutin, naringenin, quercetin, kaempferol, myricetin, cyanidin-3-glucoside, malvidin-3-glucoside, delphinidin-3-glucoside, petunidin-3-glucoside, resveratrol, oleuropein, verbascoside, and aesculetin (≥98% purity), were obtained from Extrasynthèse (Genay, France) or Sigma-Aldrich (Steinheim, Germany). All reagents and solvents were of analytical or chromatographic grade and used without further purification.

### 2.2. Experimental Design and Silage Preparation

Two experimental silages were prepared using agro-industrial by-products, primarily grape pomace (GP) and olive mill wastewater (OMWW), co-ensiled with wheat straw, cheese whey, and molasses (Table 1).

GP was obtained from a winery in Basilicata (southern Italy) after the pressing of *Vitis vinifera* L. (cv. Primitivo). OMWW was collected from the milling of *Olea europaea* L. (cv. Coratina) using a three-phase extraction system. Both by-products were transported chilled to a private farm located in southern Italy, where all subsequent silage preparation and ensiling procedures were carried out. At the farm, the by-products were thoroughly mixed with chopped wheat straw (*Triticum durum* L., 3–4 cm pieces), cheese whey (CW), and molasses (Mol) using a feed mixer wagon to prepare the experimental silages. For SIL-2, the solid-to-liquid ratio was adjusted to 60:40 following Kafantaris et al. [31], whereas SIL-1 was prepared at a 70:30 ratio (Table 1). A commercial freeze-dried inoculum of *Lactiplantibacillus plantarum* (14/DCSL CECT 4528; 250 × 10^9^ cfu g^−1^; Lactosil, CSL, Zelo Buon Persico, Lodi, Italy) was added in accordance with the manufacturer’s instructions to promote lactic acid fermentation and ensure proper stabilization of the silage mass. *Lactiplantibacillus plantarum* was selected as a homofermentative lactic acid bacterium due to its well-documented ability to rapidly dominate the epiphytic microbiota and promote fast acidification of silage [32].

### 2.3. Sampling and Sample Preparation

Silage samples were collected at multiple time points to monitor quality over storage. On day 0 (D0), immediately after silage preparation, samples were collected to represent the initial composition of each formulation. Subsequent samplings were performed at D15, D30, D60, and D150. At each sampling time point, three different bales per treatment were randomly selected. Each bale was considered an independent experimental unit, and different bales were used at each time point. From each selected bale, silage samples (~1 kg) were collected from different regions (top and bottom, approximately 30 cm from the surface, and central core) to account for within-bale variability. The subsamples collected from each bale were combined to obtain one representative sample for that bale. Each representative sample was subsequently divided into four subsamples. One subsample was used immediately for analyses on fresh matter, while the remaining three were processed for specific determinations: one was oven-dried at 60 °C, one was lyophilized, and one was stored at −20 °C until further analyses.

All analytical determinations were conducted on adequately homogenized samples.

### 2.4. Chemical Analyses

pH was measured on a 1:10 (*w*/*v*) water extract with a digital meter (SevenCompact™ S220, Mettler Toledo, Schwerzenbach, Switzerland). Buffering capacity was assessed by titration with 0.1 N NaOH as described by Playne and McDonald [33]. Ground material (1 mm) was analyzed for dry matter (DM) (method 950.46), crude protein (CP) (990.03), ether extract (920.39), and ash (920.153) according to AOAC [34]. Fiber fractions neutral detergent fiber (NDF), acid detergent fiber (ADF), and acid detergent lignin (ADL) were determined using the Van Soest method [35]. Water-soluble carbohydrates (WSC) were quantified from frozen samples (−20 °C) following Dubois et al. [36]. Ammonia nitrogen (NH_3_–N) and lactic acid were determined by colorimetric assays according to Weatherburn [37] and D’Alessandro et al. [28], respectively. For both analyses, the extraction procedures were modified in this study to optimize homogenization and sample clarification. Briefly, 500 mg of fresh silage were extracted with 4–5 mL of deionized water using an Ultra-Turrax homogenizer (IKA Werke GmbH & Co. KG, Staufen, Germany) for 3 min, followed by 10 min sonication at room temperature and two centrifugation steps (5000 and 15,000 rpm, 10 min each, 4 °C). For NH_3_–N determination, the clarified supernatant was acidified with 7.2 N H_2_SO_4_ and analyzed by the phenol–hypochlorite reaction as described by Partovi et al. [38]. For lactic acid determination, the clarified extract was diluted (1:50) with deionized water and analyzed following the colorimetric procedure reported by D’Alessandro et al. [28]. Accordingly, lactic acid was quantified using a dedicated colorimetric assay and was not included in the GC–MS analysis. Concentrations were calculated from calibration curves prepared with ammonium chloride and lactic acid standards, respectively, and expressed on a dry-matter basis.

### 2.5. Short-Chain Fatty Acids (SCFA)

The analysis of short-chain fatty acids (SCFA) was carried out according to D’Alessandro et al. [28] with modifications in the extraction procedure. Briefly, 500 mg of fresh silage was extracted with 7.5 mL of distilled water under magnetic stirring for 15 min, centrifuged for 10 min at 4200 rpm and 4 °C, and 2.5 mL of the supernatant was acidified to pH 1 with 1.5 mL of 25% H_3_PO_4_. Five milliliters of methanol were then added to the samples, and the mixture was transferred into GC vials. Extracts were analyzed immediately or stored at −80 °C until analysis.

Gas chromatography–mass spectrometry (GC–MS) analysis was performed using a Trace 1610 GC system equipped with a TriPlus™ RSH SMART autosampler (Thermo Fisher Scientific, Rodano, Milan, Italy) and a TSQ™ 9610 triple quadrupole mass spectrometer (Thermo Fisher Scientific, Austin, TX, USA). Separation was achieved on a BP21 capillary column (30 m × 0.25 mm i.d., 0.25 µm film thickness; Trajan, Ringwood, Australia). One microliter of extract was injected in split mode (75:1). The oven temperature was programmed from 40 °C (1 min) to 160 °C at 10 °C/min, with a total run time of 21 min. Helium was used as carrier gas at a constant flow of 1.2 mL/min, and the MS was operated in full-scan mode (*m*/*z* 30–450).

Quantification was performed by external calibration using a commercial SCFA standard mixture (Supelco, Bellefonte, PA, USA; TraceCERT^®^, CRM46975, 10 mM in water). Calibration curves were constructed using seven concentration levels obtained by serial dilution of the standard mixture in methanol. Owing to the different concentration ranges of individual SCFAs, separate calibration curves were applied, covering concentration ranges between 0.001 and 3 mM. Linearity was confirmed for all analytes, with coefficients of determination (R^2^) ≥ 0.99. Compound identity was confirmed by comparison with authentic standards and the NIST mass spectral library. Representative GC–MS chromatograms of SCFA silage samples are provided in the Supplementary Materials (Figures S1 and S2).

### 2.6. Lipid Extraction and FAME Derivatization

Lipids were extracted from 2 g of silage following mechanical homogenization using an Ultra-Turrax system, with a chloroform–methanol mixture (2:1, *v*/*v*) and converted to fatty acid methyl esters (FAMEs) according to Patel et al. [39], with minor adaptations for silage matrices. After phase separation and washing with phosphate buffer, the organic layer was collected, evaporated under nitrogen, and weighed to determine total lipid content. Aliquots of the lipid extract (15 mg) were transmethylated with methanolic NaOH and methanolic HCl, spiked with nonadecanoic acid (C19:0) as internal standard, and the resulting FAMEs were extracted in hexane, evaporated, and re-dissolved in 250 µL of hexane for GC–MS analysis. Quantification was performed by external calibration using a 37-component FAME mix (Supelco, Bellefonte, PA, USA). Six calibration points were prepared by serial dilution of the stock solution up to 1:32. Calibration levels were obtained through a combination of dilution factors (1:4 to 1:32) and injection volumes (1–4 µL) to cover a wide dynamic range of analyte concentrations. Each calibration point and all samples were spiked with nonadecanoic acid (C19:0) from a 1 g L^−1^ stock solution to reach a final concentration of 8 ppm after solvent evaporation and reconstitution in 250 µL of hexane. Calibration curves were constructed from the ratio of analyte to internal standard peak areas versus concentration and showed good linearity for all analytes (R^2^ > 0.99). FAME identity was confirmed by comparison of retention times and mass spectra with authentic standards and the NIST mass spectral library.

### 2.7. FAME Analysis by GC–MS

The analysis of FAME was performed according to Sánchez-Parra et al. [40] using a Trace 1610 gas chromatograph (Thermo Fisher Scientific, Rodano, Milan, Italy) coupled to a TSQ™ 9610 triple quadrupole mass spectrometer and equipped with a TriPlus™ RSH SMART autosampler (Thermo Fisher Scientific, Austin, TX, USA). Separation was achieved on a CP-Sil 88 fused silica capillary column (60 m × 0.25 mm i.d., 0.25 µm film thickness; Agilent Technologies, Santa Clara, CA, USA).

Samples (1.0 µL) were injected in split mode (5:1) at an injector temperature of 225 °C, using helium as carrier gas at a constant flow of 1.0 mL min^−1^. The oven temperature program was: initial 100 °C (2 min), ramped to 180 °C at 3 °C min^−1^ (2 min hold), then to 225 °C at 2.5 °C min^−1^ (2 min hold), for a total run time of approximately 48 min. The MS was operated in electron-impact ionization mode (EI, 70 eV) with a source temperature of 220 °C and transfer line at 230 °C, scanning the range *m/z* 45–350.

Data acquisition and processing were performed using Xcalibur software (version 4.6.67.17, Thermo Fisher Scientific, Austin, TX, USA). Representative GC–MS chromatograms obtained from FAME analyses are reported in the Supplementary Materials (Figures S3 and S4).

### 2.8. Hydrophilic Extraction

Lyophilized and finely ground silage samples (200 mg) were weighed into Eppendorf tubes and extracted according to Tuárez-García et al. [41]. Briefly, 1 mL of methanol: water (80:20, *v*/*v*) solution containing 1% formic acid was added to each sample, followed by vortex mixing for 10 s and sonication for 10 min. Samples were then centrifuged for 15 min at 4 °C at maximum speed. The supernatant was separated from the pellet, and the extraction procedure was repeated on the remaining pellet under the same conditions. The two supernatants were combined and adjusted to a final volume of 2 mL in a volumetric flask. One milliliter of the final extract was used for antioxidant activity assays, while the remaining 1 mL was used for phenolic compound identification and quantification by UHPLC-MS/MS. Total phenolic content (TPC) was determined using the Folin–Ciocalteu method [42].

### 2.9. Antioxidant Activity

Antioxidant capacity was evaluated by ABTS, DPPH, and ORAC assays following the procedure described by Tuárez-García et al. [41], using the hydrophilic extract described in Section 2.8, suitably diluted when necessary to fall within the linear range of each assay. Results were expressed as g Trolox equivalents per kg DM.

### 2.10. Polyphenolic Analysis

The analysis of phenolic compounds was carried out using a UHPLC-MS/MS system, equipped with Vanquish Flex UHPLC (Thermo Fisher Scientific, Germering, Germany) coupled to triple quadruple TSQ Fortis™ Plus (Thermo Fisher Scientific, CA, USA). Analyses were performed on the hydrophilic extracts obtained as described in Section 2.8. The separation of phenolic compounds was carried out on a Zorbax SB-C18 RRHD column (100 × 2.1 mm, i.d. 1.8 μm (Agilent, Santa Clara, CA, USA)) with a guard column of the same stationary phase maintained at 40 °C. The analytical procedure was carried out according to the method described by Pereira-Caro et al. [43]. Mass spectrometric detection was performed in negative and positive ionization modes. Compounds were identified by comparison of retention times and mass signals with those of injected analytical standards. Standard solutions of phenolic compounds were prepared in methanol:water (80:20, *v*/*v*). Stock solutions were diluted and pooled to obtain mixed standard solutions at a final concentration of 200 mg/L for each compound. Nine working solutions, ranging from 0.01 to 50 mg/L, were prepared to construct the calibration curves. Phenolic compounds were quantified based on the theoretical exact mass of their molecular ions using external calibration curves. When authentic reference standards were unavailable, quantification was performed using calibration curves of structurally related compounds. Calibration ranges were selected according to the expected concentration levels in samples and showed good linearity (R^2^ > 0.99). Detailed information on phenolic compound identification and quantification is provided in Tables S5 and S6. Representative UHPLC-MS/MS chromatograms used for polyphenol identification are reported in the Supplementary Materials (Figures S5 and S6).

### 2.11. Statistical Analysis

Data are presented as mean ± standard deviation (SD) of three independent biological replicates (bales) per treatment and sampling time. When applicable, analytical measurements were conducted in replicate and averaged prior to statistical analysis. Data were analyzed by two-way analysis of variance (ANOVA) according to the following linear model:

$$Y_{ijk}=\mu +T_{i}+D_{j}+(T\times D{)}_{ij}+\varepsilon_{ijk}$$

where $Y_{ijk}$ represents the observed value of each variable, μ  the overall mean, $T_{i\;}$ the fixed effect of treatment (SIL-1, SIL-2), $D_{j}$ the fixed effect of ensiling time (0, 15, 30, 60, 150 days), $(T\times D{)}_{ij}$ the interaction between treatment and time, and $\varepsilon_{ijk}$ the random error term associated with independent bale-level replicates.

Assumptions of ANOVA (normality and homogeneity of variance) were verified prior to analysis. When significant effects were detected, mean separation was carried out using Tukey’s HSD post hoc test.

Effect sizes were estimated using partial eta squared (η^2^p) for the two-way ANOVA factors (treatment, time, and treatment × time interaction), and eta squared (η^2^) for one-way ANOVA analyses, based on the corresponding sums of squares from the ANOVA models, to quantify the proportion of variance explained by each factor. Statistical significance was set at *p* < 0.05, *p* < 0.01, and *p* < 0.001. All analyses were performed using R software (version 3.6.3; R Core Team, Vienna, Austria).

## 3. Results

### 3.1. Chemical Composition and Fatty Acid Profile

The chemical composition of the silage mixtures was influenced by the type of treatment for DM, CP, NDF, and ADF, showing higher CP values in SIL-1, and higher dry matter (DM) and fiber fractions (NDF, ADF) in SIL-2 (Table 2). These differences were associated with large treatment effect sizes, particularly for NDF (η^2^p = 0.96), ADF (η^2^p = 0.97), and CP (η^2^p = 0.90), indicating a strong influence of silage formulation on chemical composition.

Over time, DM decreased from D0 to D30, with a slight increase at D60, and then decreased again at D150, following a similar trend observed for CP. NDF and ADF were not affected by time and remained stable throughout the entire period. Consistently, higher time-related effect sizes were observed for DM (η^2^p = 0.71) and CP (η^2^p = 0.62) compared with the other chemical composition parameters. The treatment × time interaction showed an effect (*p* < 0.05) for ADF. In SIL-1, ADF values ranged from 39 to 41% of DM, while in SIL-2, DM remained approximately 46% throughout the experimental period (Supplementary Table S1). These dynamics indicated different degradation patterns of the lignocellulosic fraction between the two experimental silages (Supplementary Table S1).

The long-chain fatty acid methyl ester (FAME) profile revealed differences between the two treatments (Table 2). In particular, SIL-1 showed significantly higher concentrations of capric, myristic, palmitoleic, and linoleic acids compared with SIL-2. Treatment exerted a strong effect on several fatty acids, with particularly high effect sizes observed for stearic (η^2^p = 1.00), capric (η^2^p = 0.98), and palmitic acids (η^2^p = 0.98). Conversely, arachidic acid was higher in SIL-2.

Ensiling time affected the concentration of most FAME (Table 2). Overall, individual fatty acids showed marked temporal variation, with high η^2^p values indicating a strong effect of ensiling time on most compounds, whereas palmitoleic acid and arachidic acid exhibited more moderate changes. Treatment × time interactions were detected for several FAMEs, including capric (C10:0), lauric (C12:0), myristic (C14:0), palmitic (C16:0), stearic (C18:0), and linoleic (C18:2), while margaric acid (C17:0) showed a weaker interaction (Supplementary Table S1). These interactions indicate that the temporal pattern of these fatty acids differed between treatments, with SIL-1 and SIL-2 showing non-parallel trends during ensiling. Overall, these results suggest distinct lipid transformation patterns between the two formulations, likely driven by differences in silage composition and microbial metabolism during storage (Supplementary Table S1).

### 3.2. Fermentative Parameters and Short-Chain Fatty Acid Profile

Fermentative parameters differed between treatments and over the ensiling period (Table 3), with high η^2^p values indicating a strong influence of both treatment and time effects, particularly for pH, buffer capacity, and WSC. SIL-1 showed lower average pH values (3.8) than SIL-2 (3.9). pH values decreased from 3.93 at D0 to 3.88 at D15, 3.82 at D30, and at D60, a slight increase was observed (3.85), whereas after 150 days (D150), the silage reached the lowest value (3.70). Overall, this pattern reflects a non-monotonic trend with minor fluctuations over time. The buffer capacity was higher in SIL-1 compared with SIL-2, indicating greater resistance to pH fluctuations in the GP-based silage. Water-soluble carbohydrate (WSC) concentration was higher in SIL-2 than in SIL-1, and this value decreased from D0 to D150. Ammonia nitrogen was higher in SIL-2 than in SIL-1, reaching a peak on D30 (74 mg/kg DM) and then declining toward the end of storage (D150; Table 3).

The treatment × time interaction affected all fermentative parameters of the silages (Supplementary Table S2). A significant interaction was also observed for pH, with all time points showing differences within each treatment in both SIL-1 and SIL-2. pH decreased from D0 to D15, D30, and D60 and subsequently increased at D150. The buffer capacity was higher in SIL-1 than in SIL-2 throughout the entire ensiling period.

Both WSC and NH_3_–N exhibited a non-linear trend over time in both silages.

The SCFA profile was influenced by both the treatment and ensiling time (Table 3), with high η^2^p values reflecting strong effects of both factors across most compounds. SIL-1 showed higher concentrations of all SCFA, except for lactic acid, which was lower than in SIL-2.

During the ensiling process, lactic acid increased over time, while individual SCFA concentrations varied in a compound-specific manner.

Acetic acid reached a pronounced peak on D15, followed by a gradual decline until D150. Propionic acid decreased sharply from D0 to near-zero values after D30, and isobutyric acid exhibited a similar decreasing trend throughout the experimental period. Butyric acid showed moderate fluctuations, increasing until D60 and then declining thereafter. Isovaleric acid remained similar on D0 and D15, whereas lower and comparable values were observed on D30, D60, and D150. Hexanoic acid displayed time-dependent differences across sampling days. Significant treatment × time interactions were observed for all SCFAs, except lactic acid (Supplementary Table S2).

### 3.3. Total Phenolic Content and Antioxidant Capacity

Total phenolic content (TPC) was higher in SIL-1 than in SIL-2 (*p* < 0.001; Table 4), with a high η^2^p value (0.93) indicating a strong influence of treatment. No significant time effect was detected, and lower η^2^p values confirmed a limited impact of the temporal factor. TPC values ranged from 2.07 g GAE/kg DM at D0 to 1.67 at D150 (Table 4). A significant treatment × time interaction was detected for TPC, with consistently higher values in SIL-1 throughout the entire ensiling period (Supplementary Table S3).

Antioxidant capacity differed between treatments in all assays. The three methodologies used, ABTS, DPPH, and ORAC, showed higher values in SIL-1 than in SIL-2 (Table 4), with η^2^p values indicating a stronger treatment effect for ABTS and DPPH compared with ORAC. Over time, ABTS activity decreased progressively from 351 to 228 g Trolox/kg DM (*p* < 0.001), supported by a relatively high η^2^p value, whereas DPPH and ORAC remained stable and showed lower η^2^p values, indicating a limited effect of time. Significant treatment × time interactions were observed for ABTS and DPPH, reflecting higher values in SIL-1 and different temporal patterns between the two silages, whereas ORAC showed a weaker interaction (*p* < 0.05) (Supplementary Table S3).

### 3.4. Polyphenolic Profile

The polyphenolic profile differed markedly between treatments and exhibited significant temporal variation during ensiling (Table 5 and Table 6), with generally high η^2^ values indicating strong time-related effects across most phenolic classes (Table 5). In SIL-1 (Table 5), flavan-3-ols increased up to D30 and then declined, with catechin as the predominant compound. (Epi)catechin exhibited a comparable temporal pattern, largely driving the evolution of this subclass over time.

All flavanones showed significant time effects (*p* < 0.001), with the exception of naringenin. Hesperetin glucoside was the main contributor at the initial sampling times and decreased during ensiling. Given its predominance, the early temporal pattern of the flavanone subclass largely reflected the trend of this compound, whereas eriodictyol-7-O-rutinoside decreased progressively until D60 and then showed a slight increase at D150.

Among flavonols, quercetin was the dominant compound and reached its maximum concentration at D150 (*p* < 0.001), whereas quercetin-3-glucoside exhibited a fluctuating trend, with a transient increase at D15 followed by a decrease at later stages (*p* < 0.001).

The flavone fraction was entirely represented by luteolin, which reached its highest concentration at D30.

All anthocyanins, except peonidin hexoside I, were significantly affected by time and showed non-linear variations during ensiling.

Hydroxybenzoic acids (gallic, 4-hydroxybenzoic, and ellagic acids) increased continuously up to D150 (*p* < 0.001), whereas hydroxycinnamic acids (caffeic, ferulic, and *p*-coumaric acids) decreased sharply during the first 30–60 days, with partial recovery toward D150 (*p* < 0.001).

Minor classes, including stilbenes (trans-resveratrol; *p* < 0.05), coumarins (aesculetin; *p* < 0.001), and other phenolics (4-vinylphenol, tryptophol, isoeugenol; *p* < 0.001), also varied during storage.

Overall, the total phenolic compound content of SIL-1 decreased from 52 mg/100 g DM at D0 to 36 mg/100 g DM at D150 (*p* < 0.01; Table 5), with a high η^2^ value (0.96) indicating a strong effect of time.

In SIL-2, all phenolic subclasses decreased markedly throughout the ensiling period (Table 6), with consistently high η^2^ values indicating strong time-related effects across all phenolic subclasses.

Flavanones (hesperetin glucoside) were detected only at D0.

Flavonols (quercetin and quercetin-3-glucoside) showed a fluctuating trend, with the highest concentration at D0 and the lowest at D150. On the other hand, the rutin progressively decreased from D0 to D30, whereas at D60 and D150, it was not detected. In contrast, flavones (luteolin, apigenin) decreased rapidly after the first 30 days and remained stable until D150, whereas chrysoeriol was detected only at D0 and D15.

Total hydroxycinnamic acids dropped markedly from D0 to D30 and partially recovered toward D150, while total hydroxybenzoic acids declined up to D30 and then showed a slight increase at later sampling times.

Secoiridoids typical of OMWW (oleuropein, oleuropein aglycone, oleacin [3,4-DHPEA-EDA], and verbascoside) were detected only at D0 and were not measurable thereafter (*p* < 0.001).

Isoeugenol decreased from D0 to D150 (*p* < 0.001).

Overall, the total phenolic content of SIL-2 declined from 22.1 to 6.3 mg/100 g DM between D0 and D150 (*p* < 0.001; Table 6), with a high η^2^ value (1.00) indicating a pronounced temporal effect.

## 4. Discussion

Silage represents a fundamental and cost-effective feed resource for ruminants, providing both energy and protein while allowing long-term preservation of forages as well as agro-industrial residues. However, silage nutritional quality and fermentation performance remain critical determinants of animal productivity and health. High-quality silage, with proper fermentation process, supports efficient rumen function, milk production, and growth [26,29,44]. In this context, the use of selected starter cultures is a key strategy to control fermentation dynamics and ensure consistent silage quality. Among lactic acid bacteria, *Lactiplantibacillus plantarum* (formerly *Lactobacillus plantarum)* is widely adopted as a starter culture in silage production due to its homofermentative metabolism, capacity for rapid lactic acid production, and effectiveness in suppressing spoilage microorganisms, thereby improving nutrient preservation and silage stability.

These properties have been extensively documented [32,45,46]. Accordingly, *Lactiplantibacillus plantarum* was selected in the present study to promote controlled fermentation in silages composed of agro-industrial substrates, where rapid stabilization is crucial for preserving functional compounds. Moreover, the present approach builds on previous investigations conducted on similar silage systems based on the same agro-industrial by-products [28,47], extending earlier laboratory-scale findings to farm-scale conditions. When agro-industrial by-products are used for ensiling, additional factors influence silage success, including their chemical composition (soluble carbohydrates, fiber fractions, and phenolic content), moisture level, and palatability. Proper dry matter adjustment and co-ensiling with complementary raw materials are therefore essential to optimize fermentation quality and feed acceptance [48,49,50].

In this study, the two silage formulations based on OMWW or GP reflected the distinct chemical characteristics of their raw matrices and confirmed the central role of by-product selection in shaping the nutritional and functional properties of the final silage. In SIL-2, higher dry matter (DM) and structural fiber contents (NDF and ADF) were consistent with the greater inclusion rate of straw (60% vs. 10% in SIL-1). Conversely, SIL-1 exhibited a higher crude protein concentration, attributable to the contribution of GP, which is typically richer in nitrogenous compounds (10–15% DM) [28,51]. These differences were further supported by high η^2^p values for DM, CP, NDF, and ADF, indicating a strong influence of treatment on silage chemical composition. These compositional values fall within ranges considered suitable for ruminant silages, ensuring appropriate retention times and stimulation of cellulolytic activity [26].

From a nutritional perspective, the higher dry matter and fiber contents of SIL-2 suggest greater structural effectiveness for rumen function, whereas the higher crude protein level in SIL-1 better contributes to meeting the protein requirements of ruminants, which are recommended at 12–16% DM for lactation or growth in cows [52].

The temporal variations in crude protein and DM content observed during ensiling were consistent with expected fermentation dynamics and microbial activity during silage storage. These processes typically lead to partial proteolysis and the conversion of soluble sugars into organic acids [23].

Considering the structural fiber parameters, such as ADF, the differences between the two silages changed throughout the ensiling process. SIL-2 consistently exhibited higher ADF values, with a slight decline over time, whereas SIL-1 remained relatively stable (Supplementary Table S1). These trends suggest distinct lignocellulosic degradation dynamics, likely linked to physicochemical properties of the respective matrices and the temporal evolution of the microbial community during fermentation [49] (Supplementary Table S1). The limited overall changes observed in crude protein and fiber fractions may reflect the primary role of *Lactiplantibacillus plantarum*, which mainly affects fermentation dynamics rather than the structural composition of the biomass [45,46].

Differences in lipid profiles between formulations can be attributed primarily to the distinct composition of the ensiled substrates. The inclusion of GP in SIL-1 likely contributed to higher concentrations of medium-chain saturated fatty acids (C10:0, C14:0) and unsaturated fatty acids, including linoleic acid (C18:2), which is abundant in grape seeds and skins [53,54]. The presence of these lipid precursors may influence both microbial activity and the biochemical pathways involved in lipid metabolism during fermentation [55]. Conversely, the relatively higher levels of arachidic acid (C20:0) detected in SIL-2 are consistent with residual lipids derived from olive processing associated with OMWW inclusion [15].

Significant treatment × time interactions for specific fatty acids, including capric (C10:0) and palmitic (C16:0) acids, suggest that compositional differences between silages were more pronounced during the initial stages of ensiling and tended to decrease or even reverse as fermentation progressed. These temporal variations likely reflected the influence of lipolytic activity and microbial biohydrogenation processes occurring during storage [56,57] (Supplementary Table S1). Overall, these compositional dynamics pointed to a qualitatively more favorable lipid profile in SIL-1, which appeared to be a more suitable substrate for balancing the dietary fiber fraction in ruminant rations (Supplementary Table S1).

Fermentative parameters represent essential indicators of silage quality and preservation potential. The decrease in pH below 4.0 within the first weeks of storage, observed in both formulations, indicated a successful fermentation process, suitable for suppressing the growth of undesirable microorganisms [23,26]. Nevertheless, slightly lower pH values and higher buffering capacity in SIL-1 suggest greater resistance to pH fluctuations, likely due to the intrinsic physicochemical properties and organic acid composition associated with the inclusion of GP.

Higher initial water-soluble carbohydrate levels in SIL-2, along with their rapid decline during early ensiling, indicate swift utilization of fermentable substrates, consistent with intense lactic acid bacterial activity [49,58]. Ammonia nitrogen concentrations peaked around D30 more markedly in SIL-2, indicating extensive protein deamination during the initial fermentation phase, followed by subsequent stabilization [23]. Overall, these dynamics aligned with those typically observed in well-preserved silages, where pH values below 4.0, progressive depletion of WSC, and controlled NH_3_–N accumulation collectively ensured proper fermentation and long-term preservation [22,27,59].

The SCFA profile further highlighted functional differences between the two silage formulations. SIL-2 displayed higher lactic acid concentrations, indicative of dominant homolactic fermentation and rapid acidification [26]. Conversely, SIL-1 showed higher proportions of acetic, propionic, and isobutyric acids, reflecting a greater contribution of heterolactic fermentation. These non-lactic acids may confer enhanced aerobic stability owing to their antifungal properties of non-lactic volatile acids [49,59].

Over time, SCFA profiles converged toward a stable fermentative state [23,27,58], with relatively high η^2^p values indicating a strong influence of ensiling time on several SCFAs. Polyphenols may also have influenced silage microbial ecology through selective antimicrobial effects against spoilage microorganisms, while promoting the establishment of specific fermentative communities [60,61]. Such interactions between microbial metabolism and phenolic composition could have contributed to the divergent fermentation trajectories observed between SIL-1 and SIL-2, particularly during the early stages of ensiling.

Total phenolic content (TPC) and antioxidant activity are key indicators of the functional quality of silages, as they reflect the presence of bioactive compounds capable of mitigating oxidative stress and supporting animal health. In silages prepared from agro-industrial by-products, both the qualitative composition and the stability of polyphenols depend on the characteristics of the starting substrate, the ensiling process, and interactions with the matrix. Monitoring TPC and antioxidant activity over time, therefore, provides insights into the preservation of these compounds and their potential nutritional and functional relevance for ruminants.

In the present study, the significantly higher TPC observed in SIL-1 compared with SIL-2 confirms the major contribution of GP as a source of bioactive phenolics. In addition, the greater stability of the TPC in SIL-1 during storage highlights the higher resilience of the polyphenolic fraction associated with this matrix. The absence of a significant main effect of time on TPC, together with a significant treatment × time interaction, suggests matrix-dependent differences in polyphenol stability during ensiling [62]. Specifically, SIL-1 showed greater stability, whereas SIL-2 exhibited an early decline in TPC. Similar patterns have been reported in polyphenol-rich silages, where phenolic compounds may undergo oxidation, partial degradation, or binding to the lignocellulosic matrix during fermentation [63]. From a functional perspective, these results indicate that GP-derived polyphenols are more effectively preserved throughout storage. In contrast, phenolics associated with OMWW appear more susceptible to early structural or chemical modifications, likely due to their different chemical nature and matrix interactions [64].

The greater stability observed in SIL-1 may also be related to microbial-mediated transformations occurring during ensiling. Lactic acid bacteria are known to possess enzymatic activities such as glycosidases and esterases that can modify polyphenols during fermentation [60]. In particular, esterases may promote the release of bound phenolic acids from the lignocellulosic matrix, while glycosidases can hydrolyze flavonoid glycosides into more bioactive aglycone forms [60,65]. These mechanisms may partially explain the persistence of flavan-3-ols and phenolic acids observed in SIL-1 throughout storage.

Antioxidant capacity represents a key functional attribute of silages, reflecting the persistence of compounds capable of mitigating oxidative stress in ruminants. In silages derived from agro-industrial by-products, antioxidant potential is largely driven by the type and stability of polyphenolic compounds during storage.

Antioxidant capacity followed trends similar to those of the TPC pattern, reinforcing the functional relevance of polyphenol preservation. Both ABTS and DPPH radical-scavenging activities were consistently higher in SIL-1, whereas ORAC remained relatively stable over time. This pattern suggests that the GP-based matrix not only provides higher antioxidant capacity but also better preserves stable antioxidant compounds during ensiling. This behavior is likely attributable to the persistence of more stable flavan-3-ols and phenolic acids, whereas more labile fractions, such as certain glycosylated flavonoids, may contribute to the observed decline in ABTS activity [43,66,67].

Previous studies have reported a strong positive correlation between TPC and antioxidant activity in silages enriched with vitivinicultural by-products [68,69], supporting the interpretation that polyphenol stability is a major driver of antioxidant potential. Importantly, silages with higher antioxidant capacity may contribute to improved oxidative status in animals by preserving or supplementing antioxidants such as α-tocopherol and β-carotene. Fermentation conditions and the use of specific lactic acid bacteria strains can further enhance antioxidant potential by protecting bioactive molecules and stimulating endogenous antioxidant enzymes, including catalase and glutathione peroxidase [70]. Conversely, the ensiling process can also result in the loss of certain antioxidants, underscoring the importance of optimized silage preparation [70].

Overall, the combination of high TPC and sustained antioxidant capacity characterizes SIL-1 as a silage with a superior functional profile throughout storage. The functional characteristics may have relevant implications for ruminant nutrition. Previous studies on silages enriched with GP and olive-derived by-products have reported improvements in systemic antioxidant status, oxidative stability, and milk fatty acid composition in lactating animals receiving bioactive silage-based diets [71]. Although the present study did not include in vivo animal evaluations, the compositional and antioxidant features observed suggest a potential application of these silages as functional feed ingredients.

During ensiling, polyphenolic compounds exhibited distinct, class-specific dynamics strongly influenced by the composition of the starting matrix. In SIL-1, flavan-3-ols, including catechin and (epi)catechin, reached peak concentrations between D15 and D30, followed by a gradual decline.

In contrast, flavonols showed distinct temporal patterns: quercetin reached high concentrations at D15 and again at D150, whereas quercetin-3-glucoside peaked at D15 and progressively declined toward D150. These observations suggest that fermentation processes may facilitate the release of bound phenolics and promote the conversion of glycosylated forms into more bioactive aglycones [72,73]. Similar transformations have been reported in fermented grape-derived matrices, where microbial and enzymatic activities enhance the release of phenolics associated with plant cell wall components [74,75]. Progressive hydrolysis of glycosylated flavonoids may further contribute to the accumulation of aglycones, which generally exhibit higher antioxidant activity and bioavailability [60,76].

Anthocyanins displayed non-linear dynamics with moderate variations over time, reflecting their inherent oxidative lability. Among hydroxybenzoic acids, gallic and ellagic acids increased progressively, indicating greater stability and a sustained contribution to the antioxidant capacity of the silage [66]. Despite an overall reduction in total polyphenol content from 52 to 36 mg/100 g DM during storage, SIL-1 retained a broad and diversified polyphenolic profile, with representative fractions persisting until the end of ensiling, highlighting its functional resilience. Differences in temporal behavior among phenolic subclasses likely reflect intrinsic chemical stability during fermentation. Anthocyanins are generally more susceptible to oxidation and degradation, whereas hydroxybenzoic acids and some flavan-3-ols exhibit greater resistance [60,77,78], explaining their persistence during prolonged storage.

In SIL-2, the significant reduction in hydroxycinnamic acids during the early stages of ensiling (D15–D30), followed by a partial recovery at D150, reflects the lower stability of this phenolic subclass. In contrast, the hydroxybenzoic acid fraction decreased sharply from D0 and remained low thereafter [64]. The rapid degradation of secoiridoids, including oleuropein and its derivatives, during the early stages of ensiling highlights a substantial structural loss typical of OMWW-based matrices [62].

The marked reduction of secoiridoids observed in SIL-2 is consistent with previous findings on olive by-products, in which oleuropein and related derivatives undergo rapid microbial and enzymatic hydrolysis during fermentation [61,79]. In fermented olive matrices, β-glucosidases and esterases produced by lactic acid bacteria and yeasts can convert oleuropein into simpler derivatives, such as hydroxytyrosol and tyrosol [79]. Although these transformation products were not specifically targeted in the present analytical approach, the observed decrease in secoiridoids likely reflects similar bioconversion processes occurring during ensiling. Overall, total polyphenol content decreased by more than 70% (from 22.1 to 6.3 mg/100 g DM) by 150 days, corresponding to a contraction of the phenolic profile and a potential reduction in the antioxidant and antimicrobial functionality of the silage [68].

From a functional perspective, the phenolic composition differed markedly between the two silage types, reflecting the chemical characteristics of their respective raw materials. SIL-1, produced from GP, was characterized by high levels of flavan-3-ols (catechin and epicatechin), flavonols (quercetin and its glycosides), proanthocyanidins, and phenolic acids (gallic and ellagic acids). These compounds are widely recognized for their strong antioxidant potential, relative stability during fermentation, and possible nutraceutical effects [67,69]. Together, they confer SIL-1 a more complex and quantitatively richer phenolic profile, consistent with the higher in vitro antioxidant activity observed.

Conversely, SIL-2, prepared with OMWW, displayed a less diverse and quantitatively lower phenolic profile. This profile was dominated by secoiridoids (oleuropein and its derivatives) and hydroxycinnamic acids, which are highly susceptible to degradation during ensiling [62,64]. The rapid loss of these compounds, evident from the early stages of fermentation, resulted in a reduction in residual phenolic content and overall antioxidant capacity relative to SIL-1.

A non-monotonic trend was observed for isoeugenol, whose concentration decreased during the early stages of ensiling (D0–D30) and subsequently increased as fermentation progressed beyond 30 days. This pattern can be plausibly explained by the interplay between microbial transformation processes and the gradual release of phenolic compounds from the silage matrix. Isoeugenol, a phenylpropanoid compound, is known to undergo microbial oxidation during anaerobic and microaerobic conditions. Previous studies have reported its conversion into intermediate compounds such as vanillin, potentially via isoeugenol-diol, followed by further transformation into metabolites including vanillic acid and guaiacol [80,81]. These pathways involving oxidative and reductive reactions mediated by bacteria and fungi may account for the initial decrease in free isoeugenol during the early fermentation phase.

In parallel, polymerization or dimerization reactions of phenylpropanoids under fermentative conditions have been described, potentially contributing to a reduction in the detectable free isoeugenol fraction. At later stages of ensiling (D30–D150), the partial increase in isoeugenol concentration may be associated with its gradual release from bound or conjugated forms within the lignocellulosic silage matrix, promoted by ongoing enzymatic activity and microbial adaptation, resulting in a dynamic balance between degradation and formation processes. Similar delayed releases of phenolic compounds during prolonged fermentation have been attributed to progressive modifications of plant cell walls and to esterase-mediated cleavage of esterified phenolics, particularly in silages rich in fibrous substrates [82,83].

It should also be noted that downstream metabolites of isoeugenol were not specifically targeted in the present analytical approach. Consequently, some transformation products may have occurred only as transient intermediates or may have been further metabolized, preventing their accumulation at detectable levels. Overall, the non-monotonic behavior of isoeugenol likely reflects a dynamic equilibrium between degradation, transformation, and gradual release processes, which are characteristic of complex, multi-substrate fermentation systems [80,81]. Collectively, these findings indicate that the choice of agro-industrial by-products plays a key role in shaping the chemical composition, affecting not only total polyphenol content but also the stability, temporal dynamics, and diversity of bioactive phenolic metabolites throughout storage. This pattern is further supported by the generally high η^2^ values, indicating strong time-related effects across most phenolic subclasses.

These compositional features ultimately contribute to the functional quality of the final silage, with potential implications for the antioxidant status of ruminants and animal-derived products, as reported for diets supplemented with agro-industrial residues [84].

Despite the promising results obtained, some limitations should be acknowledged. Although the experimental design was conducted at farm scale with biological replicates, the silage formulations investigated represent specific combinations of agro-industrial by-products selected through preliminary evaluations. Further studies, including a broader range of formulations, would help assess the general applicability of these findings. Moreover, this work primarily focused on chemical composition, fermentative parameters, and in vitro antioxidant properties, while the effects on animal performance are currently being addressed in complementary in vivo trials. Finally, the inherent variability of agro-industrial by-products may influence silage quality under different production conditions and should therefore be considered when extrapolating these results.

## 5. Conclusions

In conclusion, this study demonstrates the potential of incorporating agro-industrial by-products, such as grape pomace (GP) and olive mill wastewater (OMWW), into mixed silages including wheat straw and cheese whey as an effective strategy for valorizing high-organic-load residues within a circular bioeconomy framework. Both silage formulations achieved satisfactory fermentation quality and preservation efficiency; however, their nutritional and functional characteristics were strongly influenced by the composition of the initial substrates. The GP-based silage (SIL-1) exhibited a more favorable nutritional and biofunctional profile, characterized by higher crude protein content and a more diverse and stable polyphenolic composition, which supported greater antioxidant capacity throughout storage. In contrast, the OMWW-based silage (SIL-2) showed higher fiber content and a simpler phenolic profile, which was more susceptible to degradation during ensiling, and resulted in lower residual antioxidant activity.

By integrating farm-scale experimentation with a time-resolved evaluation of fermentative parameters, fatty acid composition, antioxidant activity, and targeted phenolic compounds, this study provides new insights into the functional potential of complex, multi-by-product silage systems under practical conditions. Overall, the results highlight that the selection and combination of agro-industrial co-products play a key role in shaping the nutritional and biofunctional quality of silages and their suitability for ruminant feeding.

Future research should focus on in vivo trials to evaluate the effects of these silages on animal performance, oxidative status, product quality, and enteric methane emissions, thereby supporting their validation as sustainable and low-emission feed resources within circular bioeconomy systems.

## Figures and Tables

**Table 1:** Experimental design and mass ratios of ingredients of the silages (% fresh matter).

Silage	Ingredients				
	WS	CW	Mol	GP	OMWW
SIL-1	10	28	2	60	0
SIL-2	60	28	2	0	10

WS = wheat straw; CW = cheese whey; Mol = molasses; GP = grape pomace; OMWW = olive mill wastewater.

**Table 2:** Chemical composition (% of dry matter) and fatty acid profile (FAME, g/kg of dry matter) of mixed by-products-based silages at different ensiling times.

Item	Treatment					Time (Days)					
	SIL-1	SIL-2	Significance	η^2^p	0	15	30	60	150	Significance	η^2^p
Chemical composition											
Dry matter	43.0 ^b^ ± 2.0	46 ^a^ ± 1.0	***	0.82	46.0 ^a^ ± 2.0	45.0 ^ab^ ± 3.0	43.0 ^b^ ± 3.0	45.0 ^ab^ ± 2.0	43.0 ^b^ ± 2.0	**	0.71
Crude Protein	8.1 ^a^ ± 1.0	5.9 ^b^ ± 1.0	***	0.90	7.5 ^a^ ± 2.0	7.2 ^ab^ ± 1.0	6.1 ^b^ ± 2.0	7.3 ^ab^ ± 1.0	6.9 ^ab^ ± 1.0	*	0.62
Ether extract	2.0 ± 0.1	2.0 ± 0.2	ns	0.009	2.1 ± 0.2	1.98 ± 0.1	1.8 ± 0.1	2.0 ± 0.04	2.0 ± 0.1	ns	0.41
Ash	8.1 ± 0.7	8.4 ± 0.4	ns	0.09	8.2 ± 0.5	8.0 ± 0.6	8.2 ± 0.3	8.4 ± 0.8	8.3 ± 0.7	ns	0.07
NDF	62.0 ^b^ ± 2.0	71.0 ^a^ ± 1.0	***	0.96	66.0 ± 6.0	66 ± 6.0	66.0 ± 4.0	66.0 ± 5.0	67.0 ± 5.0	ns	0.30
ADF	39.0 ^b^ ± 1.0	46.0 ^a^ ± 1.0	***	0.97	42.0 ± 4.0	42 ± 4.0	43.0 ± 2.0	43.0 ± 3.0	43.0 ± 3.0	ns	0.34
ADL	6.1 ± 0.3	5.8 ± 0.2	ns	0.30	5.9 ± 0.2	5.8 ± 0.5	5.8 ± 0.3	6.0 ± 0.2	6.0 ± 0.4	ns	0.19
Fatty acids											
Capric	16.0 ^a^ ± 3.0	14.0 ^b^ ± 1.0	***	0.98	15.0 ^b^ ± 1.0	19.0 ^a^ ± 3.0	15.0 ^c^ ± 1.0	14.0 ^d^ ± 1.0	14.0 ^d^ ± 2.0	***	0.99
Lauric	16.0 ± 2.0	16.0 ± 1.0	ns	0.01	17.0 ^a^ ± 2.0	16.0 ^b^ ± 1.0	15.0 ^b^ ± 2.0	14.0 ^c^ ± 1.0	16.0 ^b^ ± 1.0	***	0.93
Myristic	7.5 ^a^ ± 0.5	6.9 ^b^ ± 2.0	*	0.38	6.3 ^c^ ± 1.3	6.4 ^c^ ± 1.5	8.6 ^a^ ± 1.4	6.8 ^bc^ ± 0.8	8.1 ^ab^ ± 1.4	***	0.84
Palmitic	32.0 ^a^ ± 28.0	22.0 ^b^ ± 1.0	***	0.98	54.0 ^a^ ± 37.0	23.0 ^b^ ± 2.0	18.0 ^c^ ± 5.0	18.0 ^c^ ± 2.0	23.0 ^b^ ± 5.0	***	0.99
Palmitoleic	24.0 ^a^ ± 2.0	23.0 ^b^ ± 2.0	*	0.47	23.0 ^b^ ± 1.0	24.0 ^ab^ ± 2.0	22.0 ^b^ ± 1.0	22.0 ^b^ ± 1.0	26.0 ^a^ ± 1.0	**	0.76
Margaric	0.6 ± 0.4	0.6 ± 0.3	ns	0.03	0.8 ^b^ ± 0.09	1.01 ^a^ ± 0.1	0.2 ^d^ ± 0.1	0.4 ^c^ ±0.02	0.4 ^c^ ± 0.2	***	0.97
Stearic	0.6 ^a^ ± 0.1	0.5 ^b^ ± 0.2	***	1.00	0.7 ^a^ ± 0.03	0.5 ^e^ ± 0.3	0.6 ^b^ ± 0.02	0.5 ^c^ ± 0.01	0.5 ^d^ ± 0.02	***	1.00
Linoleic	13.0 ^a^ ± 2.0	11.0 ^b^ ± 3.0	***	0.87	14.0 ^a^ ± 1.0	10.0 ^c^ ± 6.0	13.0 ^ab^ ± 1.0	12.0 ^bc^ ± 1.0	11.0 ^c^ ± 1.0	***	0.89
Arachidic	0.7 ^b^ ± 0.3	0.9 ^a^ ± 0.1	**	0.56	0.9 ^ab^ ± 0.1	0.6 ^b^ ± 0.2	1.0 ^a^ ± 0.2	0.7 ^b^ ± 0.2	0.8 ^ab^ ± 0.1	**	0.77

SIL-1: grape pomace 60% + wheat straw 10% + cheese whey 28% + molasses 2%; SIL-2: olive mill wastewater 10% + wheat straw 60% + cheese whey 28% + molasses 2%. NDF: neutral detergent fiber; ADF = acid detergent fiber; ADL = acid detergent lignin. Values are expressed as means ± standard deviation. Different letters within the same row indicate significant differences between treatments or among ensiling times (Tukey’s test, *p* < 0.05). Significance of two-way ANOVA: * *p* < 0.05; ** *p* <0.01; *** *p* < 0.001; ns = not significant. η^2^p = partial eta squared. For interaction (treatment × time) effects, refer to Supplementary Table S1.

**Table 3:** Fermentative parameters and short-chain fatty acid (SCFA) profile of mixed by-products-based silages at different ensiling times.

Item	Treatment				Time (Days)						
	SIL-1	SIL-2	Significance	η^2^p	0	15	30	60	150	Significance	η^2^p
Fermentative characteristics											
pH	3.78 ^b^ ± 0.09	3.88 ^a^ ± 0.08	***	0.99	3.93 ^a^ ± 0.03	3.88 ^b^ ± 0.06	3.82 ^d^ ± 0.04	3.85 ^c^ ± 0.05	3.70 ^e^ ± 0.1	***	0.99
Buffer Capacity	0.27 ^a^ ± 0.05	0.13 ^b^ ± 0.04	***	1.00	0.18 ^c^ ± 0.01	0.16 ^d^ ± 0.1	0.23 ^a^ ± 0.08	0.21 ^b^ ± 0.09	0.21 ^b^ ± 0.1	***	1.00
WSC	1.68 ^b^ ± 0.7	3.34 ^a^ ± 2.7	***	0.98	4.64 ^a^ ± 3.9	2.15 ^c^ ± 0.6	2.97 ^b^ ± 0.03	1.68 ^d^ ± 0.3	1.11 ^e^ ± 1.2	***	0.98
NH_3_–N	58.0 ^b^ ± 14.0	71.0 ^a^ ± 18.0	***	0.73	70.0 ^ab^ ± 7.0	64.0 ^ab^ ± 11.0	74.0 ^a^ ± 15.0	59.0 ^b^ ± 8.0	57.0 ^b^ ± 32.0	**	0.73
Short-chain Fatty acids											
Lactic	17.0 ^b^ ± 5.0	33.0 ^a^ ± 8.0	***	0.93	16.0 ^c^ ± 8.0	22.0 ^bc^ ± 7.0	26.0 ^ab^ ± 8.0	29.0 ^a^ ± 12.0	32.0 ^a^ ± 13.0	***	0.88
Acetic	282.0 ^a^ ± 153.0	67.0 ^b^ ± 49.0	***	0.98	104.0 ^d^ ± 30.0	318.0 ^a^ ± 236.0	159.0 ^bc^ ± 168.0	169.0 ^b^ ± 104.0	121.0 ^cd^ ± 146.0	***	0.97
Propionic	2.57 ^a^ ± 2.1	0.00 ^b^ ± 0.00	***	0.97	2.97 ^a^ ± 3.44	1.65 ^b^ ±1.97	0.00 ^d^ ± 1.02	0.88 ^c^ ± 1.07	0.92 ^c^ ± 1.16	***	0.95
Isobutyric	3.45 ^a^ ± 1.5	1.04 ^b^ ± 2.2	***	0.96	4.96 ^a^ ± 0.56	2.55 ^b^ ± 2.95	0.52 ^d^ ± 2.03	1.75 ^c^ ± 1.69	1.46 ^c^ ± 0.60	***	0.98
Butyric	6.0 ^a^ ± 4.2	3.3 ^b^ ± 3.0	***	0.95	3.6 ^b^ ± 4.18	5.9 ^a^ ± 6.82	3.0 ^b^ ± 0.74	6.8 ^a^ ± 0.51	4.0 ^b^ ± 3.54	***	0.96
Isovaleric	16.6 ^a^ ± 6.0	5.9 ^b^ ± 2.3	***	0.99	16.6 ^a^ ± 11.24	14.7 ^a^ ± 5.87	8.1 ^b^ ± 4.90	9.2 ^b^ ± 4.38	7.6 ^b^ ± 4.64	***	0.97
Hexanoic	10.9 ^a^ ± 1.9	4.3 ^b^ ± 3.8	***	0.97	4.1 ^d^ ± 4.76	6.5 ^c^ ± 7.46	10.4 ^a^ ± 2.33	8.1 ^bc^ ± 2.64	9.1 ^ab^ ± 2.29	***	0.94

SIL-1: grape pomace 60% + wheat straw 10% + cheese whey 28% + molasses 2%; SIL-2: olive mill wastewater 10% + wheat straw 60% + cheese whey 28% + molasses 2%. WSC: water-soluble carbohydrates. N–NH_3_: ammonia nitrogen. Units of measurement: buffer capacity (mEq NaOH/100 g DM); water-soluble carbohydrates (WSC, g/kg DM); ammonia nitrogen (N–NH_3_, mg/kg DM); lactic acid (g/kg DM); short-chain fatty acids (acetic, propionic, isobutyric, butyric, isovaleric, and hexanoic acids; g/kg DM). Values are expressed as means ± standard deviation. Different letters within the same row indicate significant differences between treatments or among ensiling times (Tukey’s test, *p* < 0.05). Significance of two-way ANOVA: ** *p* <0.01; *** *p* < 0.001. η^2^p = partial eta squared. For interaction (treatment × time) effects, refer to Supplementary Table S2.

**Table 4:** Total phenolic content and antioxidant capacity profile of mixed by-products-based silages at different ensiling times.

Item	Treatment				Time (Days)						
	SIL-1	SIL-2	Significance	η^2^p	0	15	30	60	150	Significance	η^2^p
Total phenolic content											
	2.34 ^a^ ± 0.25	1.45 ^b^ ± 0.28	***	0.93	2.07 ± 0.20	1.81 ± 0.73	1.93 ± 0.63	1.99 ± 0.43	1.67 ± 0.66	ns	0.57
Antioxidant Capacity											
ABTS	351.0 ^a^ ± 66.0	194.0 ^b^ ± 25.0	***	0.99	351.0 ^a^ ± 132.0	253.0 ^bc^ ± 78.0	271.0 ^b^ ± 98.0	259.0 ^b^ ± 64.0	228.0 ^c^ ± 84.0	***	0.96
DPPH	348.0 ^a^ ± 36.0	211.0 ^b^ ± 48.0	***	0.96	293.0 ± 26.0	274.0 ± 110.0	302.0 ± 103.0	268.0 ± 86.0	261.0 ± 100.0	ns	0.53
ORAC	35.0 ^a^ ± 6.0	28.0 ^b^ ± 9.0	*	0.44	38.0 ± 7.0	30.0 ± 11.0	29.0 ± 12.0	32.0 ± 4.0	30.0 ± 4.0	ns	0.43

SIL-1: grape pomace 60% + wheat straw 10% + cheese whey 28% + molasses 2%; SIL-2: olive mill wastewater 10% + wheat straw 60% + cheese whey 28% + molasses 2%. Total phenolic content (TPC, Folin–Ciocalteu method) expressed as g gallic acid/kg DM; ABTS (2,2′-azino-bis(3-ethylbenzothiazoline-6-sulfonic acid) expressed as g Trolox/kg DM; DPPH (2,2-diphenyl-1-picrylhydrazyl) expressed as g Trolox/kg DM; ORAC (oxygen radical absorbance capacity) expressed as g Trolox/kg DM. Values are expressed as means ± standard deviation. Different letters within the same row indicate significant differences between treatments or among ensiling times (Tukey’s test, *p* < 0.05). Significance of two-way ANOVA: * *p* < 0.05; *** *p* < 0.001; ns = not significant. η^2^p = partial eta squared. For interaction (treatment × time) effects, refer to Supplementary Table S3.

**Table 5:** Polyphenolic profile of grape pomace-based silage (SIL-1) at different ensiling times.

Family	Compounds	Time (Days)					Significance	η^2^
		0	15	30	60	150		
Flavan-3-ols								
Catechin		0.158 ^c^ ± 0.01	0.279 ^b^ ± 0.001	0.317 ^a^ ± 0.009	0.098 ^d^ ± 0.001	0.178 ^c^ ± 0.001	***	0.99
(epi)catechin		0.095 ^c^ ± 0.01	0.177 ^b^ ± 0.002	0.206 ^a^ ± 0.001	0.055 ^d^ ± 0.001	0.094 ^c^ ± 0.001	***	0.99
Procyanidin B1		0.21 ± 0.01	0.13 ± 0.12	0.21 ± 0.01	0.16 ± 0.01	0.15 ± 0.01	ns	0.39
Procyanidin B2		0.28 ^a^ ± 0.02	0.28 ^a^ ± 0.02	0.29 ^a^ ± 0.03	0.18 ^b^ ± 0.01	0.17 ^b^ ± 0.01	**	0.93
Procyanidin C1		0.45 ^ab^ ± 0.05	0.56 ^a^ ± 0.03	0.54 ^a^ ± 0.01	0.30 ^b^ ± 0.04	0.30 ^b^ ± 0.05	**	0.94
Total		1.18 ^bc^ ± 0.05	1.43 ^ab^ ± 0.18	1.56 ^a^ ± 0.05	0.80 ^d^ ± 0.03	0.90 ^cd^ ± 0.06	**	0.95
Flavanones								
Hesperetin glucoside		0.416 ^a^ ± 0.055	0.290 ^b^ ± 0.017	0.282 ^b^ ± 0.006	0.214 ^b^ ± 0.010	0.011 ^c^ ± 0.003	***	0.98
Naringenin		0.096 ± 0.009	0.095 ± 0.002	0.094 ± 0.001	0.095 ± 0.001	0.101 ± 0.001	ns	0.41
Eriodictyol-7-O-rutinoside		0.083 ^a^ ± 0.001	0.050 ^b^ ± 0.006	0.038 ^c^ ± 0.001	0.020 ^d^ ± 0.001	0.033 ^c^ ± 0.002	***	0.99
Total		0.59 ^a^ ± 0.07	0.44 ^b^ ± 0.02	0.41 ^b^ ± 0.01	0.33 ^c^ ± 0.01	0.14 ^d^ ± 0.01	***	0.97
Flavonols								
Quercetin		0.29 ^b^ ± 0.02	0.32 ^b^ ± 0.02	0.32 ^b^ ± 0.01	0.31 ^b^ ± 0.01	0.51 ^a^ ± 0.01	***	0.99
Quercetin 3-glucoside		0.023 ^d^ ± 0.002	0.133 ^a^ ± 0.003	0.123 ^b^ ± 0.001	0.059 ^c^ ± 0.001	0.026 ^d^ ± 0.001	***	0.99
Total		0.31 ^d^ ± 0.01	0.45 ^b^ ± 0.02	0.44 ^b^ ± 0.05	0.37 ^c^ ± 0.03	0.53 ^a^ ± 0.06	***	0.98
Flavones								
Luteolin		0.82 ^c^ ± 0.01	0.58 ^d^ ± 0.02	1.16 ^a^ ± 0.01	0.92 ^b^ ± 0.04	0.57 ^d^ ± 0.01	***	0.99
Total		0.82 ^c^ ± 0.01	0.58 ^d^ ± 0.02	1.16 ^a^ ± 0.01	0.92 ^b^ ± 0.04	0.57 ^d^ ± 0.01	***	0.99
Anthocyanins								
Cyanidin-3-glucoside		0.282 ^a^ ± 0.028	0.306 ^a^ ± 0.004	0.263 ^a^ ± 0.015	0.100 ^b^ ± 0.017	0.049 ^b^ ± 0.011	***	0.98
Malvidin 3-glucoside		27.0 ^ab^ ± 2.0	29.0 ^a^ ± 4.0	22.0 ^abc^ ± 1.0	20.0 ^bc^ ± 1.0	17.0 ^c^ ± 1.0	**	0.90
Delphinidin-3-Glucoside		0.437 ^a^ ± 0.022	0.333 ^b^ ± 0.022	0.273 ^b^ ± 0.008	0.184 ^c^ ± 0.015	0.080 ^d^ ± 0.017	***	0.99
Malvidin-3,5-diglucoside		2.44 ^a^ ± 0.02	2.42 ^a^ ± 0.01	1.96 ^b^ ± 0.04	1.81 ^c^ ± 0.01	1.65 ^d^ ± 0.04	***	0.99
Peonidin hexoside I		0.71 ± 0.02	0.51 ± 0.64	0.71 ± 0.01	0.71 ± 0.01	0.53 ± 0.01	ns	0.18
Peonidin hexoside II		0.314 ^a^ ± 0.014	0.262 ^b^ ± 0.021	0.165 ^c^ ± 0.001	0.132 ^d^ ± 0.002	0.068 ^e^ ± 0.002	***	0.99
Petunidin 3-glucoside		1.82 ^b^ ± 0.01	2.52 ^a^ ± 0.17	1.99 ^b^ ± 0.02	1.90 ^b^ ± 0.01	1.23 ^c^ ± 0.01	***	0.98
Total		33.0 ^ab^ ± 2.0	35.0 ^a^ ± 4.0	28.0 ^ab^ ± 1.0	25.0 ^bc^ ± 1.0	21.0 ^c^ ± 1.0	**	0.94
Hydroxybenzoic acids								
Gallic acid		0.21 ^d^ ± 0.01	0.35 ^c^ ± 0.04	0.43 ^b^ ± 0.01	0.42 ^bc^ ± 0.01	0.97 ^a^ ± 0.01	***	0.99
4-hydroxybenzoic acid		3.30 ^b^ ± 0.2	3.40 ^b^ ± 0.1	3.60 ^b^ ± 0.1	4.20 ^a^ ± 0.1	4.60 ^a^ ± 0.1	***	0.98
Ellagic acid		0.91 ^d^ ± 0.06	1.06 ^c^ ± 0.01	1.04 ^cd^ ± 0.01	1.24 ^b^ ± 0.06	1.50 ^a^ ± 0.01	***	0.98
Total		4.40 ^d^ ± 0.12	4.80 ^c^ ± 0.04	5.10 ^c^ ± 0.05	5.80 ^b^ ± 0.01	7.10 ^a^ ± 0.03	***	0.99
Hydroxycinnamic acids								
Caffeic acid		1.85 ^a^ ± 0.03	0.52 ^c^ ± 0.04	0.34 ^d^ ± 0.01	0.33 ^d^ ± 0.01	0.70 ^b^ ± 0.01	***	0.99
Ferulic acid		2.84 ^a^ ± 0.05	1.04 ^c^ ± 0.01	1.01 ^c^ ± 0.01	0.94 ^c^ ± 0.01	1.28 ^b^± 0.01	***	0.99
p-Coumaric acid		2.08 ^a^ ± 0.05	0.68 ^c^ ± 0.02	0.57 ^d^ ± 0.01	0.67 ^cd^ ± 0.01	1.07 ^b^ ± 0.01	***	0.99
Total		6.77 ^a^ ± 0.03	2.24 ^c^ ± 0.02	1.92 ^d^ ± 0.01	1.94 ^d^ ± 0.01	3.05 ^b^ ± 0.02	***	1.00
Stilbenes								
Resveratrol (trans-)		0.58 ^a^ ± 0.01	0.56 ^ab^ ± 0.01	0.56 ^ab^ ± 0.01	0.56 ^ab^ ± 0.01	0.54 ^b^ ± 0.01	*	0.86
Total		0.58 ^a^ ± 0.01	0.56 ^ab^ ± 0.01	0.56 ^ab^ ± 0.01	0.56 ^ab^ ± 0.01	0.54 ^b^ ± 0.01	*	0.86
Coumarins								
Aesculetin		0.048 ^c^ ± 0.007	0.067 ^b^ ± 0.001	0.076 ^ab^ ± 0.001	0.083 ^a^ ± 0.002	0.071 ^ab^ ± 0.001	***	0.96
Total		0.048 ^c^ ± 0.007	0.067 ^b^ ± 0.001	0.076 ^ab^ ± 0.001	0.083 ^a^ ± 0.002	0.071 ^ab^ ± 0.001	***	0.96
Others								
4-Vinylphenol		0.53 ^a^ ± 0.02	0.39 ^b^ ± 0.01	0.40 ^b^ ± 0.01	0.40 ^b^ ± 0.01	0.40 ^b^ ± 0.01	***	0.98
Tryptophol		0.79 ^a^ ± 0.01	0.46 ^b^ ± 0.01	0.43 ^d^ ± 0.01	0.44 ^c^ ± 0.01	0.39 ^e^ ± 0.01	***	1.00
Isoeugenol		3.53 ^a^ ± 0.01	1.33 ^d^ ± 0.01	1.18 ^e^ ± 0.01	1.44 ^c^ ± 0.01	2.02 ^b^ ± 0.01	***	1.00
Total		4.80 ^a^ ± 0.1	2.20 ^d^ ± 0.1	2.00 ^e^ ± 0.1	2.30 ^c^ ± 0.1	2.80 ^b^ ± 0.1	***	1.00
Total phenolic compounds		52.0 ^a^ ± 1.0	48.0 ^ab^ ± 4.0	41.0 ^bc^ ± 1.0	38.0 ^c^ ± 1.0	36.0 ^c^ ± 1.0	**	0.96

SIL-1: grape pomace 60% + wheat straw 10% + cheese whey 28% + molasses 2%. Values are expressed as mg/100 g DM. Compounds were grouped into subclasses: flavan-3-ols, flavanones, flavonols, flavones, anthocyanins, hydroxybenzoic acids, hydroxycinnamic acids, stilbenes, coumarins, and other phenolics. Reported data represent mean values ± standard deviation. Different letters within the same row indicate significant differences among ensiling times (Tukey’s test, *p* < 0.05). Significance of one-way ANOVA: * *p* < 0.05; ** *p* < 0.01; *** *p* < 0.001; ns = not significant. η^2^ = eta squared. For detailed information on phenolic compound identification and quantification, refer to Supplementary Table S4.

**Table 6:** Polyphenolic profile of olive mill wastewater-based silage (SIL-2) at different ensiling times.

Family	Compounds	Time (Days)					Significance	η^2^
		0	15	30	60	150		
Flavanones								
Hesperetin glucoside		0.79 ^a^ ± 0.01	nd	nd	nd	nd	***	1.00
Total		0.79 ^a^ ± 0.01	nd	nd	nd	nd	***	1.00
Flavonols								
Quercetin		0.118 ^a^ ± 0.001	0.029 ^c^ ± 0.001	0.032 ^b^ ± 0.001	0.029 ^c^ ± 0.001	0.026 ^d^ ± 0.002	***	1.00
Quercetin-3-O-glucoside		0.366 ^a^ ± 0.001	0.028 ^b^ ± 0.001	0.017 ^c^ ± 0.001	0.018 ^c^ ± 0.002	0.014 ^d^ ± 0.001	***	1.00
Rutin		0.251 ^a^ ± 0.001	0.001 ^b^ ± 0.002	0.001 ^b^ ± 0.001	nd	nd	***	1.00
Total		0.736 ^a^ ± 0.001	0.058 ^b^ ± 0.001	0.050 ^c^ ± 0.001	0.047 ^d^ ± 0.001	0.040 ^e^ ± 0.001	***	1.00
Flavones								
Luteolin		1.270 ^a^ ± 0.05	0.480 ^b^ ± 0.01	0.350 ^c^ ± 0.01	0.350 ^c^ ± 0.02	0.350 ^c^ ± 0.01	***	1.00
Apigenin		0.425 ^a^ ± 0.03	0.097 ^b^ ± 0.01	0.082 ^c^ ± 0.01	0.069 ^d^ ± 0.03	0.071 ^d^ ± 0.02	***	1.00
Chrysoeriol		0.008 ^a^ ± 0.002	0.003 ^b^ ± 0.001	nd	nd	nd	***	0.99
Total		1.704 ^a^ ± 0.003	0.585 ^b^ ± 0.002	0.436 ^c^ ± 0.001	0.419 ^d^ ± 0.002	0.419 ^d^ ± 0.001	***	1.00
Hydroxycinnamic acids								
Caffeic acid		0.981 ^a^ ± 0.004	0.030 ^c^ ± 0.002	0.026 ^c^ ± 0.002	0.025 ^c^ ± 0.001	0.062 ^b^ ± 0.001	***	1.00
Chlorogenic acid		0.890 ^a^ ± 0.060	0.244 ^b^ ± 0.001	0.243 ^b^ ± 0.001	0.240 ^b^ ± 0.003	0.239 ^b^ ± 0.002	***	0.99
Caffeoylquinic acid		0.370 ^a^ ± 0.001	0.242 ^c^ ± 0.001	0.244 ^b^ ± 0.001	0.242 ^c^ ± 0.001	0.236 ^d^ ± 0.002	***	1.00
p-Coumaric acid		2.330 ^a^ ± 0.01	0.46 ^d^ ± 0.01	0.35 ^e^ ± 0.01	0.52 ^c^ ± 0.01	0.81 ^b^ ± 0.01	***	1.00
Ferulic acid		5.110 ^a^ ± 0.03	1.41 ^c^ ± 0.01	1.20 ^d^ ± 0.01	1.39 ^c^ ± 0.02	1.90 ^b^ ± 0.01	***	1.00
Total		9.70 ^a^ ± 0.08	2.40 ^c^ ± 0.01	2.10 ^d^ ± 0.01	2.40 ^c^± 0.02	3.20 ^b^ ± 0.01	***	1.00
Hydroxybenzoic acids								
3-Hydroxybenzoic acid		5.33 ^a^ ± 0.05	2.12 ^b^ ± 0.10	1.45 ^d^ ± 0.01	1.56 ^cd^ ± 0.01	1.73 ^c^ ± 0.01	***	0.99
Ellagic acid		0.458 ^a^ ± 0.005	nd	nd	nd	nd	***	1.00
Total		5.79 ^a^ ± 0.04	2.12 ^b^ ± 0.10	1.45 ^d^ ± 0.01	1.56 ^cd^ ± 0.01	1.73 ^c^ ± 0.01	***	1.00
Secoiridoids								
Oleuropein		0.008 ^a^ ± 0.001	nd	nd	nd	nd	***	0.99
Oleuropein aglycone		0.029 ^a^ ± 0.001	nd	nd	nd	nd	***	1.00
Oleacin (3,4-DHPEA-EDA)		0.036 ^a^ ± 0.001	nd	nd	nd	nd	***	1.00
Verbascoside		0.055 ^a^ ± 0.001	nd	nd	nd	nd	***	0.99
Total		0.129 ^a^ ± 0.001	nd	nd	nd	nd	***	1.00
Others								
Isoeugenol		3.30 ^a^ ± 0.09	0.15 ^cd^ ± 0.01	0.03 ^d^ ± 0.01	0.25 ^c^ ± 0.01	0.82 ^b^ ± 0.01	***	0.99
Total		3.30 ^a^ ± 0.09	0.15 ^cd^ ± 0.01	0.03 ^d^ ± 0.01	0.25 ^c^ ± 0.01	0.82 ^b^ ± 0.01	***	0.99
Total phenolic compounds		22.1 ^a^ ± 0.03	5.3 ^c^ ± 0.10	4.0 ^e^ ± 0.01	4.7 ^d^ ± 0.01	6.3 ^b^ ± 0.01	***	1.00

SIL-2: olive mill wastewater 10% + wheat straw 60% + cheese whey 28% + molasses 2%. Values are expressed as mg/100 g DM. Compounds were grouped into subclasses: flavanones, flavonols, flavones, hydroxycinnamic acids, hydroxybenzoic acids, secoiridoids, and other phenolics. Reported data represent mean values ± standard deviation. Different letters within the same row indicate significant differences among ensiling times (Tukey’s test, *p* < 0.05). Significance of one-way ANOVA: *** *p* < 0.001; nd = not detected. η^2^ = eta squared. For detailed information on phenolic compound identification and quantification, refer to Supplementary Table S5.

## Data Availability

The original contributions presented in this study are included in the article/Supplementary Material. Further inquiries can be directed to the corresponding authors.

## Supplementary Materials

The following supporting information can be downloaded at: https://www.mdpi.com/article/10.3390/antiox15060692/s1. Table S1: ANOVA interactions between treatment and time factors and their effects on chemical composition and fatty acid profile values in silage samples; Table S2: ANOVA interactions between treatment and time factors and their effects on fermentative characteristics and short-chain fatty acid profile values in silage samples; Table S3: ANOVA interactions between treatment and time factors and their effects on total phenolic content and antioxidant profile values in silage samples; Table S4: Tentative identification of phenolic compounds in SIL-1 by HPLC-MS/MS; Table S5: Tentative identification of phenolic compounds in SIL-2 by HPLC-MS/MS; Figure S1: Representative GC–MS chromatogram of SCFA in SIL-1 at 60 days of ensiling; Figure S2: Representative GC–MS chromatogram of SCFA in SIL-2 at 60 days of ensiling; Figure S3: Representative GC–MS chromatogram of FAME in SIL-1 at 60 days of ensiling; Figure S4: Representative GC–MS chromatogram of FAME in SIL-2 at 60 days of ensiling; Figure S5: Representative UHPLC–MS/MS SRM chromatogram of phenolic compounds in SIL-1 at 60 days of ensiling; Figure S6: Representative UHPLC–MS/MS SRM chromatogram of phenolic compounds in SIL-2 at 60 days of ensiling.

## Abbreviations

The following abbreviations are used in this manuscript:

ABTS	2,2′-Azino-bis(3-ethylbenzothiazoline-6-sulfonic acid)
ADF	Acid Detergent Fiber
ADL	Acid Detergent Lignin
ANOVA	Analysis of Variance
AAPH	2,2′-Azobis(2-amidinopropane) dihydrochloride
CP	Crude Protein
CW	Cheese Whey
DM	Dry Matter
DPPH	2,2-Diphenyl-1-picrylhydrazyl
ESI	Electrospray Ionization
FAME	Fatty Acid Methyl Ester
GAE	Gallic Acid Equivalent
GC	Gas Chromatography
GC–MS	Gas Chromatography–Mass Spectrometry
GP	Grape Pomace
HPLC	High-Performance Liquid Chromatography
MRM	Multiple Reaction Monitoring
MS	Mass Spectrometry
NDF	Neutral Detergent Fiber
NH_3_–N	Ammonia Nitrogen
OMWW	Olive Mill Wastewater
ORAC	Oxygen Radical Absorbance Capacity
SCFA	Short-Chain Fatty Acid
TPC	Total Phenolic Content
UHPLC	Ultra-High-Performance Liquid Chromatography
WS	Wheat Straw
