# Supplementary material for: Bioactive Silages from Agro-Industrial By-Products Based on Grape Pomace or Olive Mill Wastewater for Ruminants: Evolution of Phenolic Profiles, Antioxidant Activity, and Fatty Acid Composition
Source: Antioxidants (Basel). 2026 May 30;15(6):692. doi: 10.3390/antiox15060692 (PMC13295314; doi:10.3390/antiox15060692)
Supplement: Supplementary file 1 [file antioxidants-15-00692-s001.zip › antioxidants-4298301-supplementary.pdf]

Title: Bioactive Silages from Agro-Industrial By-Products Based on Grape Pomace or Olive Mill Wastewater for Ruminants: Evolution of Phenolic Profiles, Antioxidant Activity, and Fatty Acid Composition

Authors: R.S. Dibenedetto, M. Sánchez-Parra, J.L. Ordóñez-Díaz, A. Di Luca, G. Martemucci, J.M. Moreno-Rojas, A.G. D'Alessandro

Data were analyzed using a two-way ANOVA model including treatment, time, and their interaction as fixed effects. Post hoc comparisons were performed using Tukey's HSD test when significant effects were detected. Significance levels were set at $P < 0.05$

Supporting Table S1. ANOVA interactions between treatment and time factors and their effects on chemical composition and fatty acid profile values in silage samples.

Item	Interaction										*Signifi-cance*	η²p
	**1 x 0**	**1 x 15**	**1 x 30**	**1 x 60**	**1 x 150**	**2 x 0**	**2 x 15**	**2 x 30**	**2 x 60**	**2 x 150**		
Chemical composition												
Dry matter	45.0 ± 1.0	43.0 ± 1.0	43.0 ± 1.0	42.0 ± 3.0	41.0 ± 1.0	47.0 ± 1.0	47.0 ± 1.0	47.0 ± 1.0	45.0 ± 1.0	44.0 ± 1.0	ns	0.15
Protein	9.0 ± 0.1	8.2 ± 0.4	8.6 ± 0.8	8.1 ± 0.3	6.7 ± 0.7	6.1 ± 0.5	6.2 ± 0.5	6.0 ± 0.2	5.7 ± 0.8	5.6 ± 0.6	ns	0.39
Ether extrac	2.02 ± 0.03	1.94 ± 0.09	2.12 ± 0.01	2.08 ± 0.01	1.84 ± 0.15	2.14 ± 0.40	2.02 ± 0.06	1.88 ± 0.03	2.01 ± 0.01	1.84 ± 0.04	ns	0.29
Ash	8.0 ± 0.6	7.7 ± 0.7	8.3 ± 0.4	8.3 ± 1.3	8.0 ± 0.9	8.4 ± 0.2	8.3 ± 0.5	8.5 ± 0.3	8.2 ± 0.6	8.3 ± 0.7	ns	0.05
NDF	61.0 ± 1.0	60.0 ± 1.0	62.0 ± 2.0	63.0 ± 2.0	62.0 ± 3.0	71.0 ± 1.0	71.0 ± 1.0	69.0 ± 1.0	71.0 ± 1.0	70.0 ± 1.0	ns	0.42
ADF	39.0 ^{b} ± 1.0	38.0 ^{b} ± 1.0	41.0 ^{b} ± 1.0	40.0 ^{b} ± 1.0	40.0 ^{b} ± 1.0	46.0 ^{a} ± 1.0	46.0 ^{a} ± 1.0	45.0 ^{a} ± 1.0	46.0 ^{a} ± 1.0	46.0 ^{a} ± 1.0	*	0.63
ADL	6.0 ± 1.0	5.9 ± 1.0	6.3 ± 1.0	6.2 ± 1.0	6.0 ± 1.0	5.8 ± 1.0	5.8 ± 1.0	5.8 ± 1.0	5.9 ± 1.0	5.5 ± 1.0	ns	0.11
Fatty acids												
Capric	16.0 ^{b} ± 1.0	22.0 ^{a} ± 1.0	14.0 de ± 1.0	13.0 e ± 1.0	16.0 b ± 1.0	15.0 c ± 1.0	16.0 b ± 1.0	14.0 cd ± 1.0	14.0 cd ± 1.0	13.0 e ± 1.0	***	0.98
Lauric	19.0 a ± 1	16 b ± 1	12 c ± 1	16 b ± 1	15 b ± 1	16 b ± 1	16 b ± 1	15 b ± 1	16 b ± 1	16 b ± 1	***	0.93

Myristic	7.3 ^{bc} ± 0.9	7.5 ^{bc} ± 0.6	8.0 ^{ab} ± 0.1	7.5 ^{bc} ± 0.8	7.4 ^{bc} ± 0.1	5.3 ^{c} ± 0.3	5.3 ^{c} ± 1.1	5.6 ^{c} ± 0.3	8.7 ^{ab} ± 0.2	9.8 ^{a} ± 0.3	***	0.86
Palmitic	86.0 ^{a} ± 1.0	24.0 ^{b} ± 2.0	13.0 ^{c} ± 1.0	24.0 ^{b} ± 1.0	14.0 ^{c} ± 1.0	22.0 ^{b} ± 2.0	21.0 ^{b} ± 1.0	22.0 ^{b} ± 1.0	21.0 ^{b} ± 1.0	23.0 ^{b} ± 1.0	***	0.99
Palmitoleic	23.0 ± 1.0	26.0 ± 1.0	23.0 ± 1.0	27.0 ± 1.0	22.0 ± 1.0	23.0 ± 2.0	23.0 ± 1.0	21.0 ± 1.0	25.0 ± 1.0	23.0 ± 1.0	ns	0.41
Margaric	0.86 ^{a} ± 0.06	1.02 ^{a} ± 0.05	0.53 ^{bc} ± 0.04	0.40 c ± 0.01	nd	0.74^{ab} ± 0.06	1.00 ^{a} ± 0.21	0.33 ^{c} ± 0.01	0.43 ^{c} ± 0.01	0.41 ^{c} ± 0.01	**	0.79
Stearic	0.66 ^{b}± 0.01	0.72 ^{a} ± 0.02	0.56 ^{f} ± 0.01	0.51 ^{h} ± 0.01	0.57 ^{e} ± 0.03	0.66 ^{c} ± 0.01	0.22 ^{j} ± 0.01	0.52 g ± 0.02	0.50 ^{i} ± 0.02	0.60 ^{d} ± 0.02	***	1.00
Linoleic	15.0 ^{ab} ± 1.0	16.0 ^{a} ± 1.0	12.0 ^{bc} ± 1.0	11.0 ^{c} ± 1.0	13.0 ^{bc} ± 1.0	14.0 ^{ab} ± 1.0	5.0 ^{d} ± 1.0	11.0 ^{c} ± 1.0	11 .0^{c} ± 1.0	13.0 ^{b}c ± 1.0	***	0.96
Arachidic	0.86 ± 0.09	0.38 ± 0.03	0.52 ± 0.06	0.67 ± 0.06	1.07 ± 0.18	0.90 ± 0.17	0.79 ± 0.13	0.80 ± 0.21	0.99 ± 0.13	1.02 ± 0.05	ns	0.48

Interaction: treatment (1, 2) x ensiling time (0, 15, 30, 60, 150 days), where: 1 = SIL-1: grape pomace 60% + wheat straw 10% + cheese whey 28% + molasses 2%; 2 = SIL-2: olive mill wastewater 10% + wheat straw 60% + cheese whey 28% + molasses 2%. Significance of the ANOVA interaction: *P < 0.05; **P < 0.01; ***P < 0.001; ns = not significant. nd= not detected. η^2p = partial eta squared. Reported data represent mean values ± standard deviation. Means with different letters in the same row indicate significant differences ($P < 0.05$) ns = not significant. 1 = SIL-1; 2 = SIL-2. NDF = Neutral detergent fiber; ADF = Acid detergent fiber; ADL = Acid detergent lignin.

Supporting Table S2. ANOVA interactions between treatment and time factors and their effects on fermentative characteristics and short-chain fatty acid profile values in silage samples.

Item	Interaction										Significance	η^2p
	1 x 0	1 x 15	1 x 30	1 x 60	1 x 150	2 x 0	2 x 15	2 x 30	2 x 60	2 x 150		
Fermentative characteristcs												
pH	3.9 ^{c} ± 0.1	3.8 ^{e} ± 0.1	3.8 ^{f} ± 0.1	3.6 ^{h} ± 0.1	3.7 ^{g} ± 0.1	4.0 ^{a} ± 0.1	3.9 ^{b} ± 0.1	3.9 ^{d} ± 0.1	3.7 ^{g} ± 0.1	3.9 ^{c} ± 0.1	***	0.93
Buffer Capacity	0.18 ^{e} ± 0.01	0.26 ^{d} ± 0.01	0.28^{c} ± 0.01	0.29 ^{b} ± 0.01	0.33 ^{a} ± 0.01	0.18 ^{f} ± 0.01	0.06 ^{j} ± 0.01	0.14 ^{g} ± 0.01	0.13^{h} ± 0.01	0.13 ^{i} ± 0.01	***	1.00
WSC	1.26 ^{de} ± 0.1	2.68 ^{c} ± 0.2	1.67^{d} ± 0.03	0.84 ^{e} ± 0.1	1.95 ^{d} ± 0.0[illegible]	8.03 ^{a} ± 0.5	1.61 ^{d} ± 0.01	1.6 ^{d} ± 0.05	1.38^{de}± 0.04	3.98^{b} ± 0.2	***	0.99
NH_3–N	74.0 ^{b} ± 6.0	73.0 ^{bc} ± 2.0	46.0 ^{d} ± 5.0	51.0 ^{cd} ± 6.0	47.0 ^{d} ± 2.0	66.0 ^{bcd} ± 7.0	54.0 ^{bcd} ± 5.0	72.0 ^{bc} ± 1.0	63.0^{bcd}±11.0	102.0 ^{a} ± 7.0	***	0.91
Short-chain Fatty acids												
Lactic	10.0 ± 2.0	16.0 ± 2.0	23.0 ± 1.0	22.0 ± 1.0	15.0 ± 1.0	22.0 ± 1.0	28.0 ± 2.0	36.0 ± 4.0	42.0 ± 8.0	37.0 ± 1.0	ns	0.50
Acetic	79.0 ^{de} ± 1.0	520.0 ^{a} ± 49.0	314.0^{b}±36.0	211.0^{c} ± 1.0	286.0^{bc} ± 1.[illegible]	129.0^{d} ± 10.0	115.0^{d} ± 12.0	25.0 ^{e} ± 1.0	31.0 ^{e} ± 1.0	33.0 ^{e} ± 2.0	***	0.96
Propionic	5.947 ^{a} ± 0.3	3.297 ^{b} ± 0.9	1.757^{c} ± 0.1	1.843^{c}± 0.2	nd	nd	nd	nd	nd	nd	***	0.95
Isobutyric	4.70 ± 0.3 ^{ab}	5.09^{a} ± 0.3	3.50 ^{bc} ± 0.2	2.92 ^{c} ± 0.3	1.04^{d} ± 0.01	5.21^{a} ± 0.8	nd	nd	nd	nd	***	0.95
Butyric	7.2^{b} ± 0.1	11.8^{a} ± 0.9	7.4 ^{b} ± 0.2	3.8 ^{c} ± 0.7	nd	nd	nd	6.3 ^{b} ± 0.6	4.2 ^{c} ± 0.3	6.1 ^{b} ± 0.2	***	0.99
Isovaleric	26.3 ^{a} ± 1.7	19.7 ^{b} ± 1.1	13.4 ^{c} ± 1.0	11.4 ^{cd} ± 0.7	12.0 ^{cd} ± 0.9	6.9 ^{ef} ± 0.5	9.6 ^{de} ± 0.4	4.9 ^{f} ± 0.3	3.9 ^{f} ± 0.2	4.1 ^{f} ± 0.9	***	0.92
Hexanoic	8.3 ^{cd} ± 0.1	12.9 ^{a} ± 0.2	9.9 ^{abc} ± 1.7	11.4 ^{ab} ± 0.4	12.1 ^{a} ± 1.3	nd	nd	6.3 ^{d} ± 0.1	6.9 ^{cd} ± 0.1	8.6 ^{bcd} ± 1.2	***	0.91

Interaction: treatment (1, 2) x ensiling time (0, 15, 30, 60, 150 days), where: 1 = SIL-1: grape pomace 60% + wheat straw 10% + cheese whey 28% + molasses 2%; 2: = SIL-2: olive mill wastewater 10% + wheat straw 60% + cheese whey 28% + molasses 2%. Significance of the ANOVA interaction: ***$P < 0.001$; ns: not significant. nd= not detected. Reported data represent mean values ± standard deviation. η^2p = partial eta squared. Means with different letters in the same row indicate significant differences ($P < 0.05$). ns = not significant. 1 = SIL-1; 2 = SIL-2. WSC: water-soluble carbohydrates. NH_3–N: ammonia nitrogen.

Supporting Table S3. ANOVA interactions between treatment and time factors and their effects on total phenolic content and antioxidant profile values in silage samples.

Item	Interaction										Significance	η^2p
	1 x 0	1 x 15	1 x 30	1 x 60	1 x 150	2 x 0	2 x 15	2 x 30	2 x 60	2 x 150		
Total phenolic content												
	2.23 ^{a} ± 0.16	2.44 ^{a} ± 0.20	2.52 ^{a} ± 0.23	2.01 ^{ab} ± 0.27	2.49 ^{a} ± 0.11	1.92 ^{ab} ± 0.05	1.19 ^{c} ± 0.04	1.45 ^{bc} ± 0.04	1.34 ^{bc} ± 0.20	1.37 ^{bc} ± 0.22	*	0.67
Antioxidant profile												
ABTS	466.0 ^{a} ± 4.0	320 ^{bc} ± 1.0	343.0 ^{b} ± 13.0	283.0^{cd}± 22.0	342.0 ^{b} ± 26	237.0^{de} ± 2.0	185.0^{f} ± 4.0	174.0 ^{f} ± 13.0	174.0 ^{f} ± 9.0	200 .0^{ef} ± 4.0	***	0.83
DPPH	294.0^{bc}± 37.0	369.0 ^{ab} ± 3.0	357.0^{ab}± 10.0	335.0^{ab} ± 3.0	386.0 ^{a} ± 20	292.0^{bc} ± 25.0	179.0^{d}± 16.0	179.0 ^{d} ± 16.0	187.0 ^{d} ± 5.0	218.0^{cd} ± 34.0	***	0.85
ORAC	35.0 ^{a} ± 7.0	39.0 ^{a} ± 4.0	42.0 ^{a} ± 3.0	32.0^{a} ± 2.0	28.0^{a}± 7.0.0	41.0^{a} ± 7.0	22.0^{a} ± 10.0	22.0 ^{a} ± 3.0	27.0 ^{a} ± 3.0	29.0 ^{a} ± 1.0	*	0.62

Interaction: treatment (1, 2) x ensiling time (0, 15, 30, 60, 150 days), where: 1 = SIL-1: grape pomace 60% + wheat straw 10% + cheese whey 28% + molasses 2%; 2: = SIL-2: olive mill wastewater 10% + wheat straw 60% + cheese whey 28% + molasses 2%. Significance of the ANOVA interaction: *P < 0.05; ***P < 0.001. η^2p = partial eta squared. Reported data represent mean values ± standard deviation. Means with different letters in the same row indicate significant differences ($P < 0.05$). ns = not significant. 1 = SIL-1; 2 = SIL-2. ABTS: 2,2′-azino-bis(3-ethylbenzothiazoline-6-sulfonic acid; DPPH: 2,2-diphenyl-1-picrylhydrazyl; ORAC: oxygen radical absorbance capacity.

Supporting Table S4. Tentative identification of phenolic compounds in SIL-1 by HPLC-MS/MS.

RT (min)	Compounds	Chemical Formula	Main fragment (m/z)-	Production(s)	Collision energy (V)	SIMI	Standard curve
11.4	Catechin	C15H14O6	289.00	124.883/ 203.05/ 245.217	18.18/ 16.6/ 11.66	1	Catechin
2.17	(epi)catechin	C15H14O6	289.07	109.083/ 151	24/ 22	1	(epi)catechin
10.9	Procyanidin B1	C30H26O12	577.14	289.05/ 407.05/ 425.05	22.97/ 22.62/ 14.24	1	Procyanidin B1
11.8	Procyanidin B2	C30H26O12	577.14	289.05/ 407.05/ 425.05	22.97/ 22.62/ 14.24	1	Procyanidin B2
12.3	Procyanidin C1	C45H38O18	865.2	407.05/ 577.133/ 695.133	40.16/ 17.03/ 22.19	1	Procyanidin C1
3.56	Hesperetin glucoside	C22H24O11	463.12	163.883/ 285.883/ 301.05	43.24/ 32.57/ 14.17	1	Hesperetin glucoside
7.73	Naringenin	C15H12O5	273.08	90.883/ 146.967/ 152.883	41.59/ 20.83/ 21.9	1	Naringenin
2.54	Eriodictyol-7-O-rutinoside	C27H32O15	595.17	287.06/ 151.00 / 135.05	20/ 20/ 20	1	Eriodictyol-7-O-rutinoside
6.55	Quercetin	C15H10O7	301.04	120.967/ 151.05/ 179.05	26.48/ 21.04/ 17.39	1	Quercetin
3.56	Quercetin-3-O-glucoside	C21H20O12	463.09	270.883/ 299.967/ 300.967	42.88/ 26.05/ 20.68	1	Quercetin-3-O-glucoside
6.38	Luteolin	C15H10O6	285.04	133.05/ 150.967/ 199.05	33.86/ 25.41/ 24.41	1	Luteolin
13,6	Cyanidin-3-glucoside	C21H21O11+	449.11*	212.967/ 240.883/ 286.967	54.41/ 51.69/ 21.9	1	Cyanidin-3-glucoside
13,5	Delphinidin-3-Glucoside	C21H21O12+	465.1*	228.967/ 256.967/ 302.967	55.27/ 47.46/ 21.97	2	Cyanidin-3-glucoside
13,9	Malvidin-3,5-diglucoside	C29H35O17+	655.19*	314.967/ 330.967/ 493.05	66.66/ 33.57/ 19.47	1	Malvidin-3,5-diglucoside
1.99	Malvidin-3-Glucoside	C23H25O12+	493.13*	286.967/ 314.967/ 330.967	49.9/ 49.9/ 21.47	1	Malvidin-3-Glucoside
1.96	Peonidin hexoside I	C22H23O11+	463.12*	228.967/ 285.967/ 300.967	64.15/ 41.74/ 21.83	2	Cyanidin-3-glucoside
4.32	Peonidin hexoside II	C22H23O12+	463.12*	228.967/ 285.967/ 300.967	64.15/ 41.74/ 21.83	2	Cyanidin-3-glucoside
1.46	Petunidin-3-Glucoside	C22H23O12+	479.12*	202.883/ 244.883/ 316.967	65.65/ 60.5/ 21.76	2	Cyanidin-3-glucoside
2.1	Gallic acid	C7H6O5	169.01	[illegible].05/ 96.883/ 106.8/ 124.967	[illegible].98/ 17.17/ 23.83/ 14.88	1	Gallic acid
10.9	4-Hydroxybenzoic acid	C7H6O3	137.02	44.883/ 93.05/ 136.55	21.26/ 11.3/ 10	1	4-Hydroxybenzoic acid

3.48	Ellagic acid	C14H6O8	301.00	184.967/ 201.05/ 229.05	29.13/ 30.35/ 26.91	1	Ellagic acid
12	Caffeic acid	C9H8O4	179.03	89.05/ 107.133/ 135.05	31.93/ 21.97/ 14.6	1	Caffeic acid
3.94	Ferulic Acid	C10H10O4	193.05	133.967/ 149.05/ 177.967	15.31/ 10.3/ 12.02	1	Ferulic Acid
13.4	p-Coumaric acid	C9H8O3	163.04	91.133/ 93.05/ 119.05	28.77/ 30.85/ 14.45	1	p-Coumaric acid
12.3	Resveratrol (trans-)	C14H12O3	229.09	106.967/ 118.967/ 134.967	23.12/ 16.46/ 15.03	1	Resveratrol (trans-)
12.1	Aesculetin	C9H6O4	177.02	105.034/ 133.03	20/ 20	2	Caffeic acid
13.4	4-Vinylphenol	C8H8O	119.05	91.055/ 93.035	20/ 20	2	Gallic acid
15.4	Tryptophol	C10H11N	162.01	144.08	20	2	Gallic acid
13.4	Isoeugenol	C10H12O2	163.08	118.99/ 148.05	20/ 20	2	p-Coumaric acid

SIL-1: grape pomace 60% + wheat straw 10% + cheese whey 28% + molasses 2%.

RT: Retention time. * These compounds were analysed with positive ionization mode. 1Metabolite Standards Initiative metabolite identification (MSIMI) levels. Reference compounds were available for all compounds identified at MSIMI level 1. Compounds at the MSIMSI level 2 were tentatively identified.

Supporting Table S5. Tentative identification of phenolic compounds in SIL-2 by HPLC-MS/MS.

RT (min)	Compounds	Chemical Formula	Main fragment (m/z)-	Production(s)	Collision energy (V)	MSIMI	Standard curve
13.5	Hesperetin glucoside	$C_{22}H_{24}O_{11}$	463.12	163.88/ 285.88/ 301.05	43.24/ 32.57/ 14.17	1	Hesperetin glucoside
16.5	Quercetin	$C_{15}H_{10}O_{7}$	301.04	151.05/ 179.05/ 120.967	21.04/ 17.39/ 26.48	1	Quercetin
13.5	Quercetin-3-O-glucoside	$C_{21}H_{20}O_{12}$	463.09	270.88/ 299.97/ 300.97	42.88/ 26.05/ 20.68	1	Quercetin-3-O-glucoside
13.2	Rutin	$C_{27}H_{30}O_{16}$	609.15	255.05/ 270.97/ 300.13	56.77/ 57.56/ 37.08	1	Rutin
16.5	Luteolin	$C_{15}H_{10}O_{6}$	285.04	133.05/ 150.97/ 199.05	33.86/ 25.41/ 24.41	1	Luteolin
17.8	Apigenin	$C_{15}H_{10}O_{5}$	269.046	93.0346/ 117.035/ 151.004/ 225.056	20/ 20/ 20/ 20	1	Apigenin
16.4	Chrysoeriol	$C_{16}H_{12}O_{6}$	299.056	153.02/ 163.004/ 206.994/ 271.025	20/ 20/ 20/ 20	2	Luteolin
11.9	Caffeic acid	$C_{9}H_{8}O_{4}$	179.03	89.05/ 107.13/ 135.05	31.93/ 21.97/ 14.6	1	Caffeic acid
11.4	Chlorogenic acid	$C_{16}H_{18}O_{9}$	353.09	84.967/ 161.05	41.95/ 20.68	1	Chlorogenic acid
12.1	Caffeoylquinic acid	$C_{16}H_{18}O_{9}$	353.09	84.967/ 161.05	41.95/ 20.68	2	Chlorogenic acid
13.4	p-Coumaric acid	$C_{9}H_{8}O_{3}$	163.04	91.13/ 93.05/ 119.05	28.77/ 30.85/ 14.45	1	Coumaric acid
13.9	Ferulic Acid	$C_{10}H_{10}O_{4}$	193.05	133.97/ 149.05/ 177.97	15.31/ 10.3/ 12.02	1	Ferulic Acid

11	3-Hydroxybenzoic acid	$C_7H_6O_3$	137.02	44.88/ 93.05/ 136.55	21.26/ 11.3/ 10	1	3-ydroxybenzoic acid
16.6	Ellagic acid	$C_{14}H_6O_8$	301	184.97/ 201.05/ 229.05	29.13/ 30.35/ 26.91	1	Ellagic acid
15.1	Oleuropein	$C_{25}H_{32}O_{13}$	539.177	89.0248/ 275.091/ 307.082/ 377.124	20/ 20/ 20/ 20	1	Oleuropein
16.9	Oleuropein aglycone	$C_{19}H_{22}O_8$	377.17	241/ 275/ 307/ 345	20/ 20/ 20/ 20	2	Oleuropein
16.7	Oleacin (3,4-DHPEA-EDA)	$C_{17}H_{20}O_6$	319.119	111/ 153/ 199	20/ 20/ 20	1	Oleacin (3,4-DHPEA-EDA)
13.5	Verbascoside	$C_{29}H_{36}O_{15}$	623.198	161.02/ 461.16	20/ 20	2	Oleuropein
13.3	Isoeugenol	$C_{10}H_{12}O_2$	163.08	118.99/ 148.05	20/ 20	2	Coumaric acid

SIL-2: olive mill wastewater 10% + wheat straw 60% + cheese whey 28% + molasses 2%.

RT: Retention time.1 Metabolite Standards Initiative metabolite identification (MSIMI) levels. Reference compounds were available for all compounds identified at MSIMI level 1. Compounds at the MSIMSI level 2 were tentatively identified.

Figure S1. Representative GC–MS chromatogram of SCFA in SIL-1 at 60 days of ensiling.

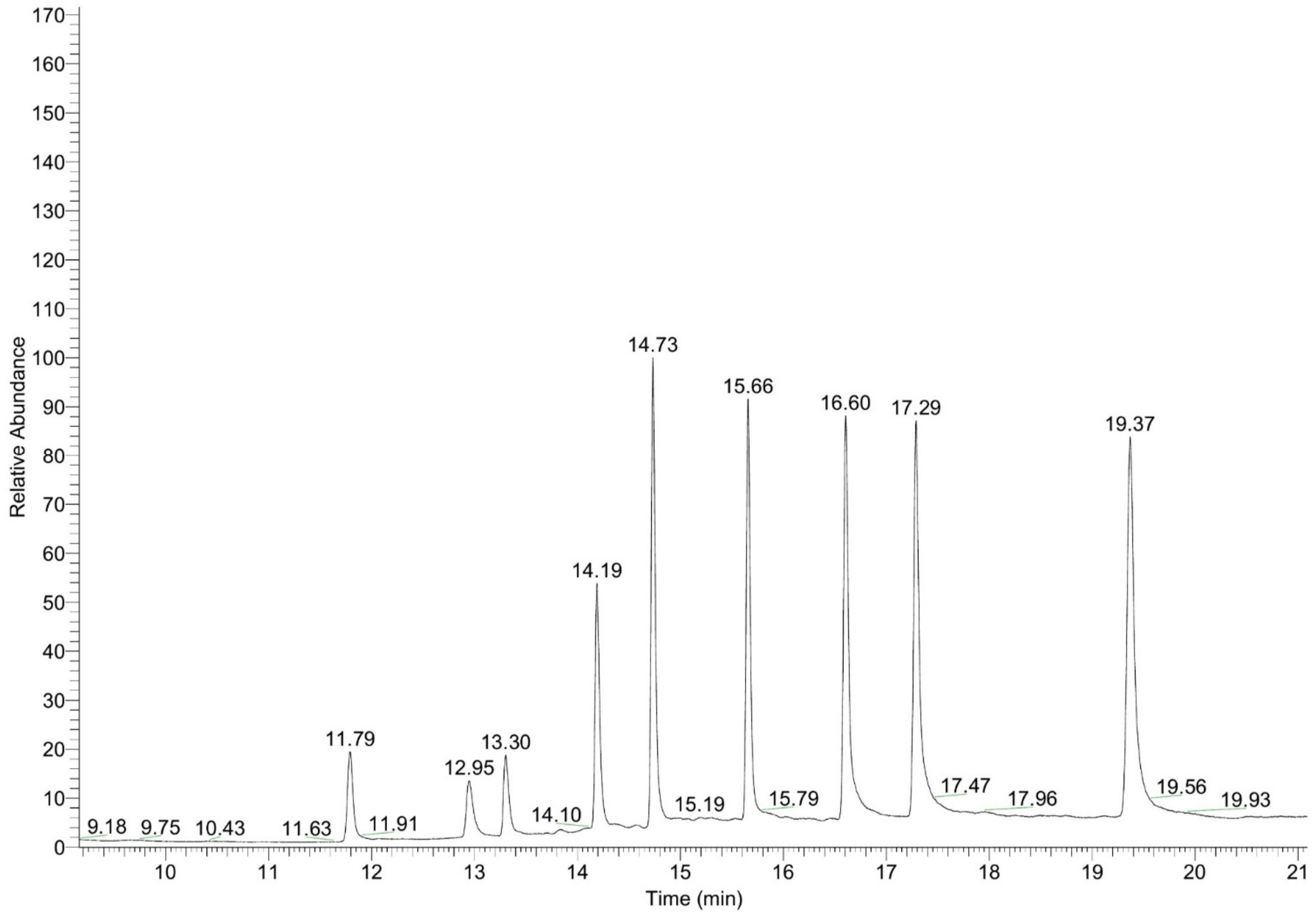

SIL-1: grape pomace 60% + wheat straw 10% + cheese whey 28% + molasses 2%.

SCFA profile detected in SIL-1 at 60 d of ensiling. Identification based on RT (retention time; min): acetic acid (11.79), propionic acid (12.95), isobutyric acid (13.30), butyric acid (14.19), isovaleric acid (14.73), valeric acid (15.66), isocaproic acid (16.60), hexanoic acid (17.29), and n-heptanoic acid (19.37).

Figure S2. Representative GC–MS chromatogram of SCFA in SIL-2 at 60 days of ensiling.

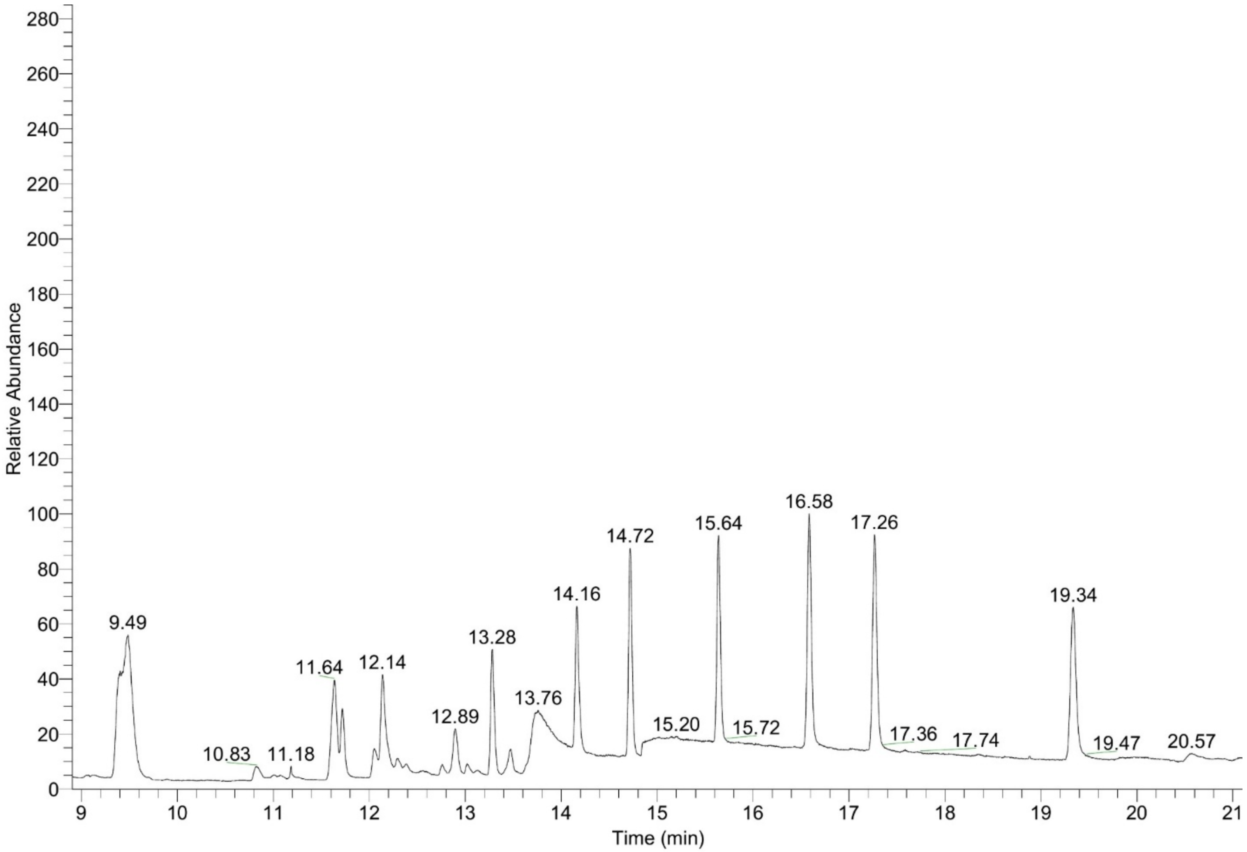

SIL-2: olive mill wastewater 10% + wheat straw 60% + cheese whey 28% + molasses 2%.

SCFA profile detected in SIL-2 at 60 d of ensiling. Identification based on RT (retention time; min): acetic acid (11.64), propionic acid (12.14), isobutyric acid (13.28), butyric acid (14.16), isovaleric acid (14.72), valeric acid (15.64), isocaproic acid (16.58), hexanoic acid (17.26), and n-heptanoic acid (19.34).

Figure S3. Representative GC–MS chromatogram of FAME in SIL-1 at 60 days of ensiling.

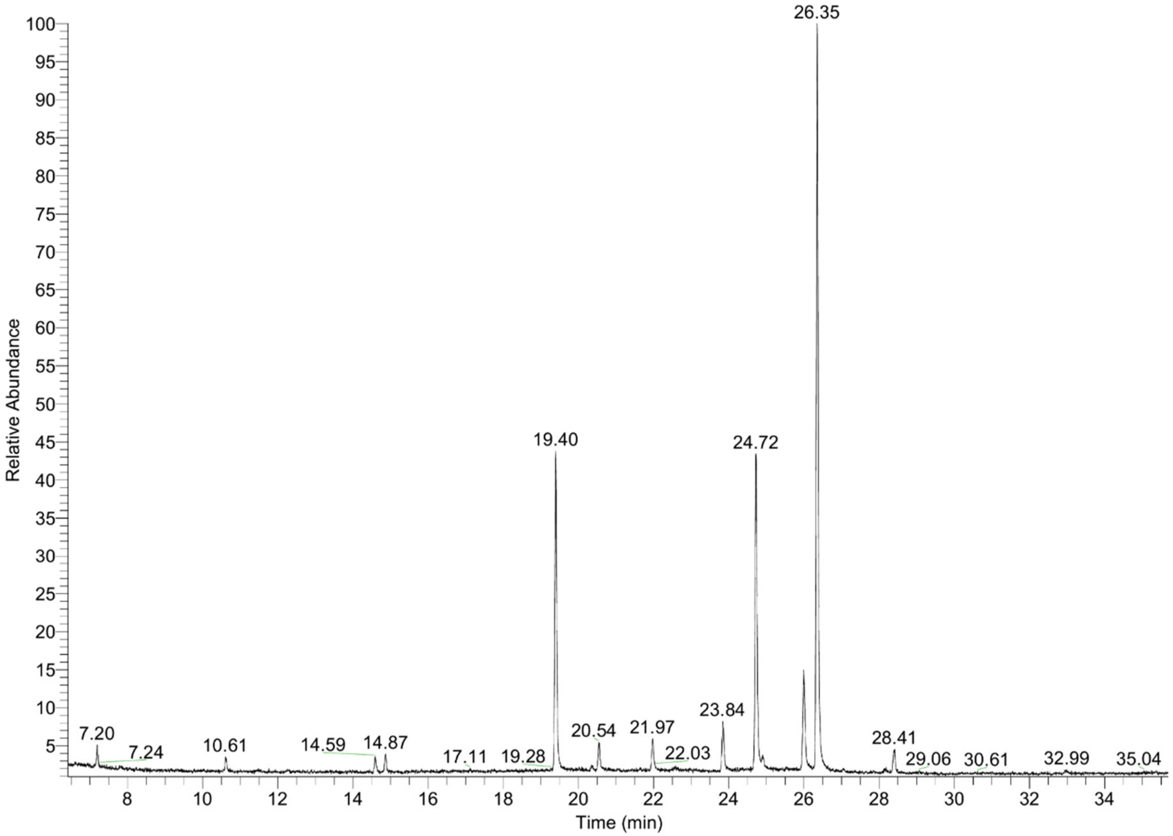

SIL-1: grape pomace 60% + wheat straw 10% + cheese whey 28% + molasses 2%.

FAME profile detected in SIL-1 at 60 d of ensiling. Identification based on RT (retention time; min) of compounds included in the Supelco 37 FAME standard mixture and quantified in the study: capric acid (7.20), lauric acid (10.61), myristic acid (14.87), palmitic acid (19.40), palmitoleic acid (20.54), margaric acid (21.97), stearic acid (23.84), linoleic acid (24.72), and arachidic acid (28.41). Internal standard (methyl nonadecanoate, C19:0 methyl ester) at 26.35 min showed good reproducibility and no interference with target FAMEs.

Figure S4. Representative GC–MS chromatogram of FAME in SIL-2 at 60 days of ensiling.

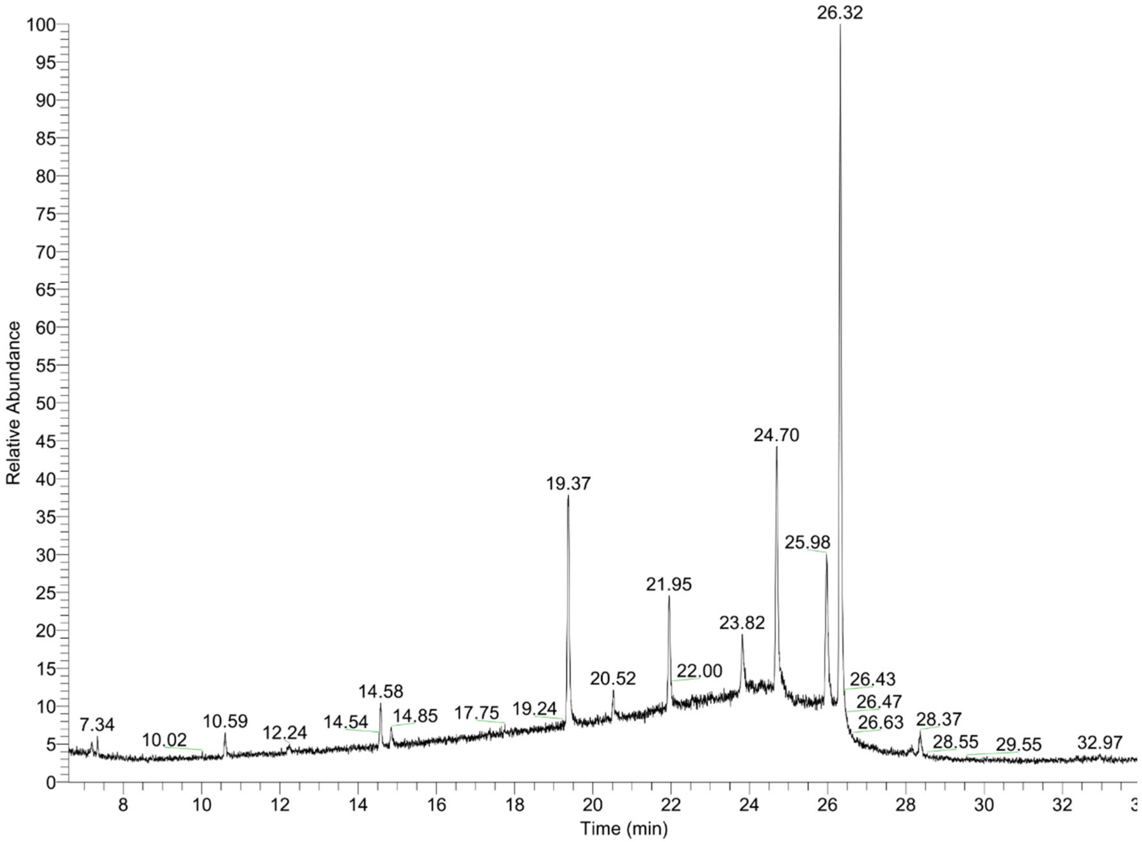

SIL-2: olive mill wastewater 10% + wheat straw 60% + cheese whey 28% + molasses 2%.

FAME profile detected in SIL-2 at 60 d of ensiling. Identification based on RT (retention time; min) of compounds included in the Supelco 37 FAME standard mixture and quantified in the study: capric acid (7.34), lauric acid (10.59), myristic acid (14.58), palmitic acid (19.37), palmitoleic acid (20.52), margaric acid (21.95), stearic acid (23.82), linoleic acid (24.70), and arachidic acid (28.37). Internal standard (methyl nonadecanoate, C19:0 methyl ester) at 26.35 min showed good reproducibility and no interference with target FAMEs.

Figure S5. Representative UHPLC–MS/MS SRM chromatogram of phenolic compounds in SIL-1 at 60 days of ensiling.

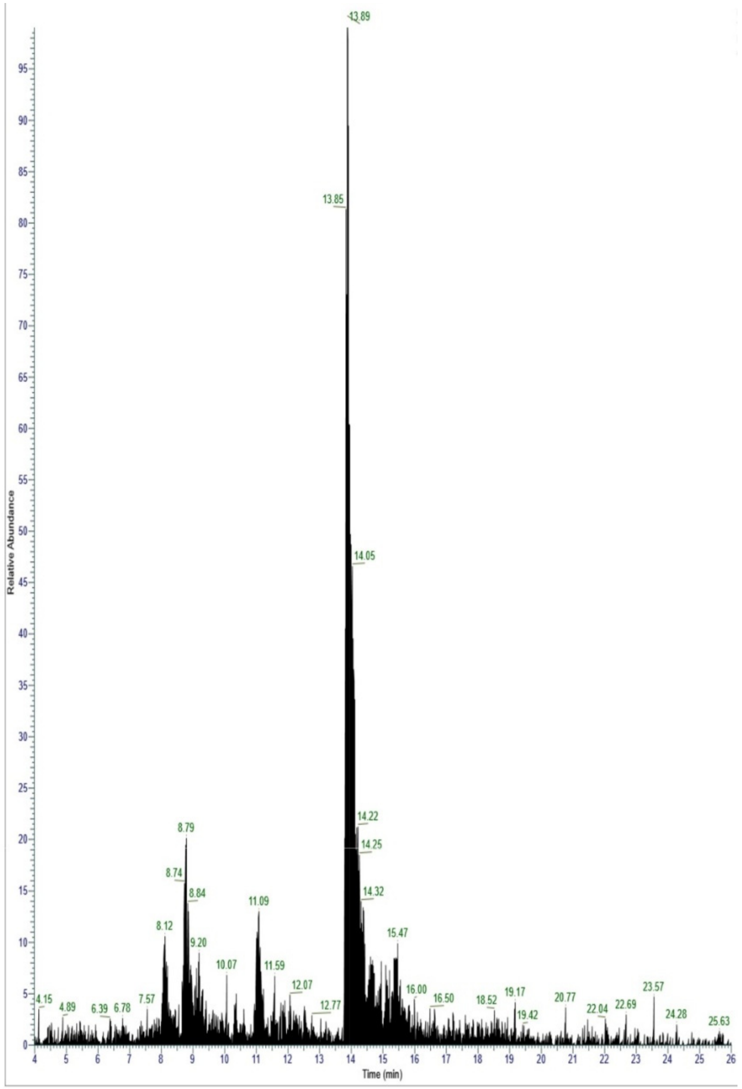

SIL-1: grape pomace 60% + wheat straw 10% + cheese whey 28% + molasses 2%.

Figure S6. Representative UHPLC–MS/MS SRM chromatogram of phenolic compounds in SIL-2 at 60 days of ensiling.

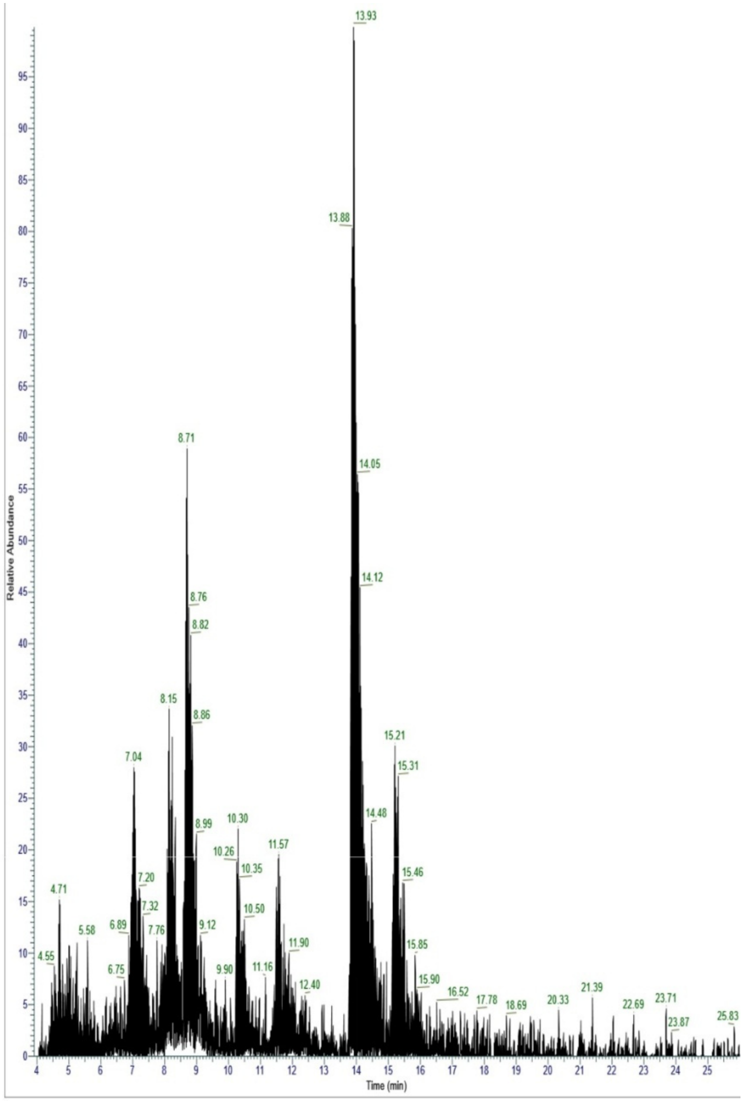

SIL-2: olive mill wastewater 10% + wheat straw 60% + cheese whey 28% + molasses 2%.
